# The interaction between inflammation and estrogen in adenomyosis : from molecular mechanisms to therapeutic strategies

**DOI:** 10.1007/s00281-026-01077-w

**Published:** 2026-06-08

**Authors:** Feng Li, Huai Jian Wang, Yu Zhang, Feng Juan Nan, Li Xu, Zhi Yong Liu, Wei Shi

**Affiliations:** 1 First Clinical Medical College, Shandong University of Traditional Chinese Medicine, Jinan, 250014 China; 2 Shandong Provincial Prison Hospital, Jinan, 250014 Shandong China; 3 Department of Gynecology, Shandong University of Traditional Chinese Medicine Affiliated Hospital, Jinan, 250014 China; 4 Department of reproductive medicine, Shandong University of Traditional Chinese Medicine Second Affiliated Hospital, Jinan, 250001 China; 5 Central Laboratory, Shandong University of Traditional Chinese Medicine Affiliated Hospital, Jinan, 250014 China

**Keywords:** Inflammation, Estrogen, Vicious cycle, Adenomyosis, Clinical management

## Abstract

Adenomyosis is a common and difficult disease in gynecology. Its main clinical manifestations include uterine hypertrophy, excessive menstruation, pelvic pain and infertility, which have a serious impact on women’s health. Seriously affects women’s health. At present, the pathogenesis of this disease is not clear; however, the increase in local estrogen and the occurrence of an inflammatory response are widely believed to be closely related to the development of adenomyotic lesions. Although progress has been made in understanding how estrogen and inflammation play their respective roles in adenomyosis, the relationship between estrogen and inflammation and its impact on adenomyosis are not fully understood. In this work, we summarize the effects of estrogen and inflammation on the occurrence and development of adenomyosis, including the involved biological processes and related signal transduction mechanisms, as well as the targeted drugs for clinical treatment, and further summarize the interactions between the two in adenomyosis as well as the available evidence for clinical applications. This review helps to broaden our understanding of the role of the vicious inflammatory‒estrogen cycle in the pathogenesis and pathophysiology of adenomyosis, and it provides new ideas for the treatment of uterine adenomyosis.

**Graphical Abstract:**

The vicious cycle of inflammation and estrogen promotes the development of adenomyosis.

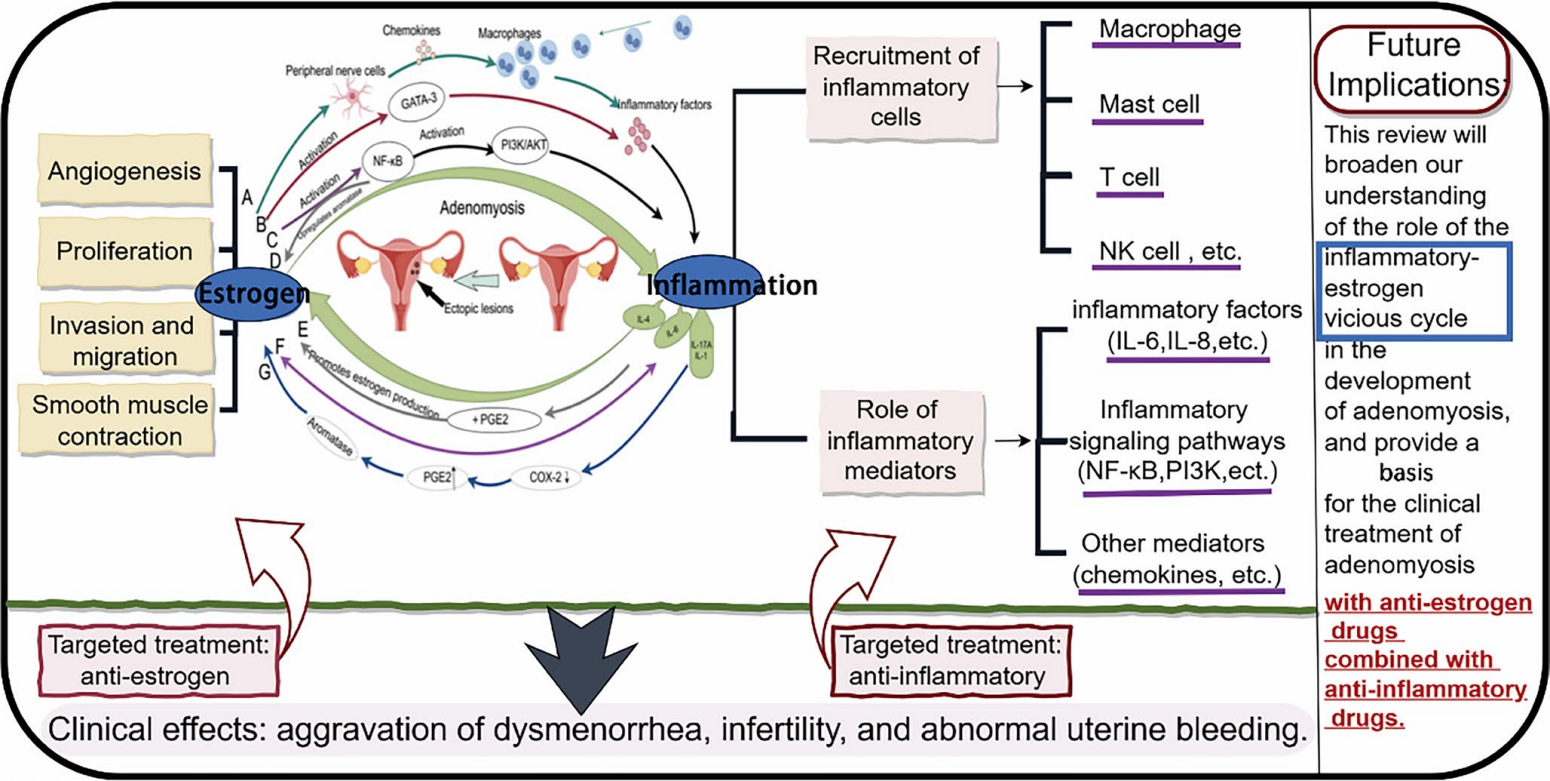

## Introduction

**Fig. 1:**
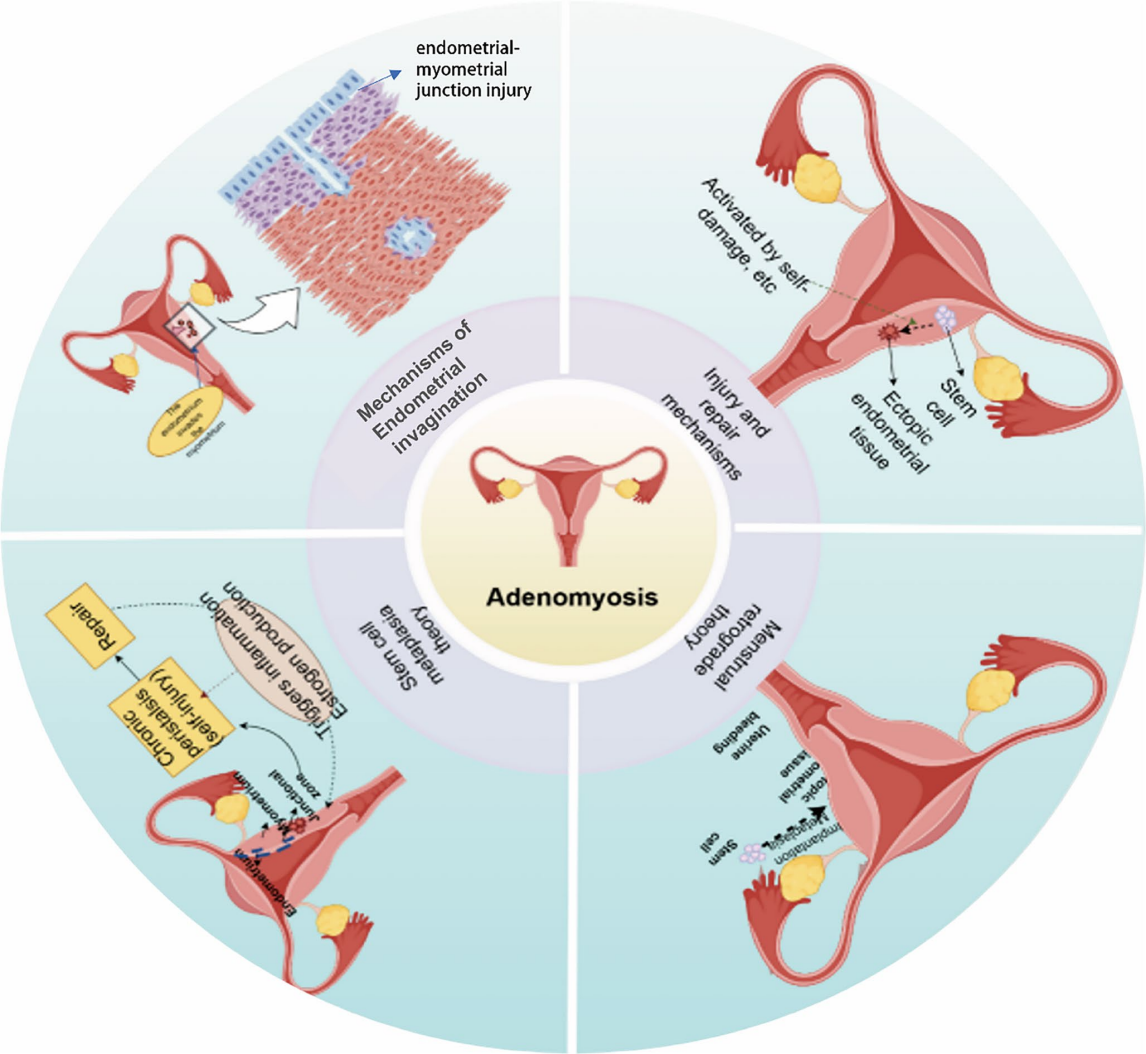
There are four theories concerning the pathogenesis of adenomyosis. (**A**) The endometrial invagination mechanism. (**B**) The mechanisms of tissue injury and repair. (**C**) The stem cell metaplasia theory. (**D**) The menstrual retrograde theory. This figure was created by Figdraw

Adenomyosis is a common gynecological disease, with a prevalence of 20.9% to 34% [1–5]. It exhibits clinical manifestations such as uterine hypertrophy, excessive menstruation, pelvic pain and infertility [6, 7]. Additionally, adenomyosis is characterized by the presence of abnormal endometrial epithelial cells and stromal fibroblasts in the myometrium and the proliferation and hypertrophy of surrounding smooth muscle cells, thereby leading to diffuse uterine enlargement [8]. Microscopically, nonneoplastic ectopic endometrial glands and stroma are surrounded by hypertrophic and proliferative myometrium [9, 10]. However, the pathogenesis of adenomyosis is not yet clear. At present, the widely studied pathogenesis mainly includes : the endometrial invagination mechanism [11–14];the mechanism of tissue injury and repair [15, 16]; the stem cell metaplasia theory [17, 18];the menstrual retrograde theory [14]. (Fig. 1) Adenomyosis is often associated with endometriosis. The characteristics of these two diseases are the presence of endometrial glands and stroma outside the normal position. Endometriosis and adenomyosis are estrogen-dependent diseases, usually accompanied by inflammatory processes. Both estrogen abnormalities and inflammatory processes are essential for the establishment and growth of ectopic endometrium [19].

The theory of endometrial invagination mainly involves structural changes in the junctional tissue between the endometrium and the myometrium. The influence of high estrogen status causes cohesion of smooth muscle cells, thus providing an optimal condition for the endometrium to sink, moreover, high estrogen levels reduce the degree of apoptosis of ectopic endometrial cells and promote their proliferation, thus resulting in the development of adenomyosis [20–24]. The mechanism of tissue damage and repair is mainly due to the chronic self-injury state that is caused by endometrial peristalsis, which elicits an inflammatory response, activates interleukin-1 (IL-1) and induces cyclooxygenase-2 (COX-2) to promote prostaglandin production, which in turn activates steroidogenic acute regulatory protein (STAR) and the P450 aromatase, thereby resulting in a local high estrogen environment. Estrogen can cause endometrial peristaltic hyperfunction and increase the inflammatory response [25–30]. The activation of endometrial stem cells or stem cells deposited by retrograde menstruation is closely related to uterine injury. Endometrial stem cells or stem cells deposited by retrograde menstruation are activated after endometrial–myometrial junction injury, which may lead to abnormal alterations in the stem cell niche, allowing their differentiating progeny to move toward the myometrium rather than the endometrial functional area [13, 18, 26]. It suggests that the pathogenesis of adenomyosis is closely related to estrogen and inflammatory responses, and there is an interaction between inflammation and estrogen in adenomyosis, which together promotes the development of the disease. At present, the clinical drugs that have been reported to treat adenomyosis mainly include nonsteroidal anti-inflammatory drugs and gonadotropin-releasing hormone agonists, which can relieve adenomyosis; however, their curative effects are limited. For example, nonsteroidal anti-inflammatory drugs are effective for treating menstrual pain caused by adenomyosis, but their therapeutic effect with respect to abnormal bleeding is not as good as that of hormone therapy. Additionally, drugs that can maintain estrogen levels are not ideal for pain relief [7, 31–34]. Therefore, the combination of estrogen inhibition and anti-inflammatory methods may achieve better therapeutic effects than single treatment does, and this article summarizes the existing evidence to provide reliable evidence for the clinical use of estrogen inhibition and anti-inflammatory methods in adenomyosis.

## Localized estrogen abnormalities promote the progression of adenomyosis

Adenomyosis is an estrogen-dependent disease [35] that is related to the biological effects of estrogen and its receptor binding to the response elements of the target gene in the nucleus, thereby initiating the ‘genomic effect’ to induce the transcription of the target gene [36]. The increased level of estrogen in the endometrium enhances the growth ability of the myometrium and ectopic tissue in the junctional zone of endometrial infiltration, which leads to excessive growth and invasion of endometrial stromal cells and promotes the epithelial-mesenchymal transition of endometrial epithelial cells and angiogenesis [10, 35, 37–43]. Studies have shown that abnormal estrogen-related biological effects caused by the overexpression of estrogen receptors and aromatase in adenomyosis are closely related to the pathogenesis of adenomyosis [36, 44]. Aromatase is a type of cytochrome P450 enzyme system. Moreover, it can synthesize estrogen by catalyzing testosterone, androstenedione and other components, thus directly affecting the expression level of estrogen.

### Abnormal synthesis of estrogen in adenomyosis

An examination of the activity of aromatase and estrone sulfatase in the ectopic endometrium revealed that estrogen is synthesized in the endometrial tissue of women with adenomyosis and may affect the occurrence and development of adenomyosis [45]. Studies have shown that women with adenomyosis had the highest menstrual blood estradiol levels, which were higher than those in the normal and endometriosis groups [46]. Polymorphisms in genes that lead to increased estrogen production, such as the expression of aromatase and COX-2, and the catechol-O-methyltransferase (COMT) 158G/A gene, which leads to decreased estrogen metabolism, are associated with an increased risk of developing adenomyosis [47–52]. Therefore, excessive estrogen is considered to be due to the overexpression of local aromatases and the reduction in local estrogen metabolism in the eutopic and ectopic endometria of patients with adenomyosis. Kitawaki et al. reported that aromatase is not present in the endometria of disease-free uteri but is observed in the ectopic endometria of adenomyosis patients [53]. This enzyme aromatizes circulating androgens into estradiol, promotes estrogen biosynthesis and increases estrogen bioavailability [53]. In addition, studies have shown that the presence of complete aromatase genes and aromatase activity are essential for the growth of ectopic uterine tissue in a mouse model of endometriosis [54]. Therefore, data suggests that aromatase should be used as a preliminary screening method for adenomyosis [55, 56].

COX-2 is overexpressed in the eutopic endometrium and ectopic endometrium of patients with adenomyosis [57], and the local high estrogen state of patients with adenomyosis stimulates the activity of COX-2, thereby promoting prostaglandin synthesis [58]. Increased prostaglandins upregulate STAR and aromatase to increase estrogen synthesis [59]. Therefore, COX-2 can promote the high estrogen state caused by adenomyosis.

### Estrogen participates in multiple biological processes of adenomyosis

#### Estrogen promotes angiogenesis

**Fig. 2:**
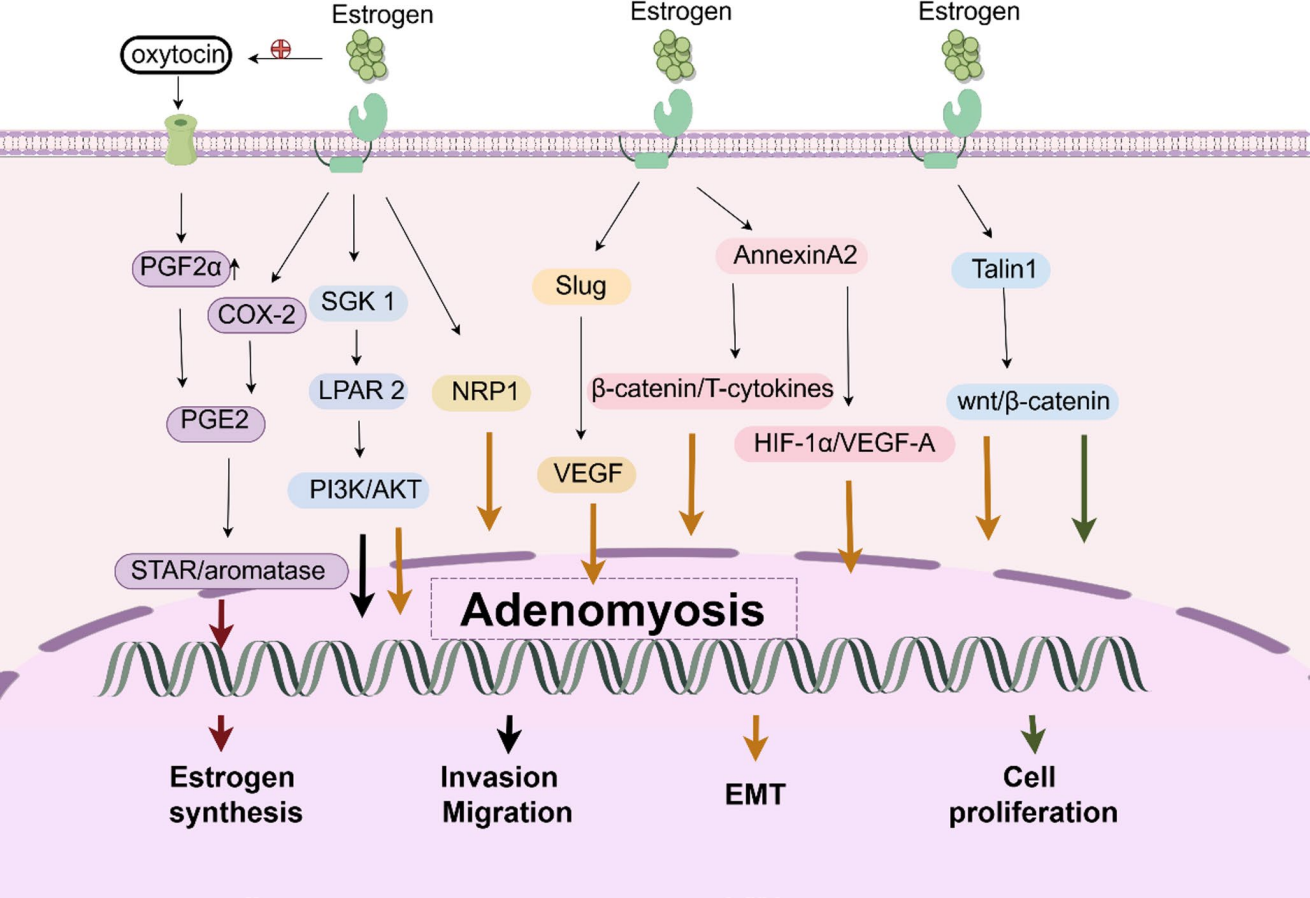
The related signaling pathways by which estrogen affects the pathogenesis of adenomyosis. Estrogen leads to the migration, invasion and proliferation of endometrial stromal cells through a variety of signaling pathways and induces epithelial-mesenchymal transition of endometrial stromal cells, which can lead to adenomyosis. This figure was created by Figdraw. *PGF2α* prostaglandin F2α, *COX-2* cyclooxygenase-2, *PEG2* prostaglandin E2, *STAR* steroidogenic acute regulatory protein, *SGK1* serum and glucocorticoid-induced kinase 1, *LPAR2* lysophosphatidic acid receptor 2, *PI3K/AKT* phosphatidylinositol 3 kinase/protein kinase B, *NRP1* neuropilin 1, *VEGF* vascular endothelial growth factor, *HIF-1α* hypoxia inducible factor-1α, *EMT* endometrial epithelial-mesenchymal transition

Estrogen stimulation can promote the proliferation of the deep perivascular matrix in adenomyosis [60]. Estradiol activates the Slug- vascular endothelial growth factor (VEGF) axis in endometrial epithelial cells and promotes angiogenesis in vascular endothelial cells [43]. Slug is a zinc finger transcription factor and an important inducer of the epithelial-mesenchymal transition [61]. Moreover, VEGF is a major angiogenic factor that plays a role in mediating angiogenesis during the development of adenomyosis [62]. Previous studies have shown that estrogen can upregulate Slug in adenomyosis to induce the epithelial-mesenchymal transition, which subsequently produces VEGF to promote angiogenesis [43]. (Fig. 2).

Zhou et al. reported that estrogen can significantly upregulate the expression of annexin A2 in the endometrium. The overexpression of annexin A2 has been confirmed to exist in the ectopic lesions of human adenomyosis. Moreover, the high expression of annexin A2 can not only induce epithelial-mesenchymal transition by changing cell structure and function to mesenchymal-like cells via β-catenin/T cytokine signal transduction but also enhance the angiogenesis ability of human endometrial cells via the HIF-1α/VEGF-A pathway [63].

Talin1 can also synergize with estradiol to induce angiogenesis and endometrial stromal cell proliferation in human adenomyosis by triggering the Wnt/β-catenin pathway [64–66]. Talin1 is a macromolecular protein that mainly functions as a key regulator of integrin activation. Previous studies have demonstrated that Talin1-dependent integrin activation can regulate VE-cadherin localization and endothelial cell barrier function, which are essential for angiogenesis and development [67, 68]. In addition, mutations in the Wnt/β-catenin pathway can lead to the abnormal activation of epithelial-mesenchymal transition and elicit the occurrence of adenomyosis. For instance, Wang et al. discovered in an in vitro experiment that Talin1 induced the epithelial-mesenchymal transition of human endometrial cells and the migration and invasion of ectopic endometrial epithelial cells, achieving this through the activation of the classical β-catenin pathway [65].

#### Effects of estrogen on the proliferation, invasion and migration of ectopic endometrial cells

Serum and glucocorticoid-induced kinase 1 (SGK1) is an important Akt-independent mediator of the PI3K signaling pathway. Studies have shown that SGK1 is an additional PI3K effector and plays a key role in the PI3K pathway [69]. Additionally, FGF18 is a member of the FGF family, which is located on chromosome 14p11. FGF18 plays a major role in the manipulation of tumor cell growth and invasion [70, 71]. Estrogen can directly act on nearby endometrial stromal cells, studies have shown that estrogen can induce FGF18 to promote the proliferation of endometrial cancer cells and transmit proliferation signals via the AKt pathway [72]. In addition, estradiol affects the abnormal activation of the PI3K/AKT signaling pathway related to tumor cell invasion and migration in most tumors by upregulating SGK1 in endometrial cells [69, 73, 74]. This upregulation results in increased expression of N-cadherin, matrix metalloproteinase 2, matrix metalloproteinase 9 and other proteins, thereby promoting the migration and invasion of human interstitial endometrial stromal cells and the epithelial-mesenchymal transition of endometrial cells [75, 76].

Neuropilin 1 (NRP1) is a transmembrane glycoprotein that is involved in the occurrence and metastasis of various cancer cells [77, 78]. Previous studies have shown that NRP1 can be targeted by miR-124-3p. Moreover, miR-124-3p downregulates the expression of NRP1 in adenomyosis and reduces the migratory activity of endometrial cells. Estrogen can also promote the migration and epithelial-mesenchymal transition of endometrial stromal cells by upregulating the expression of NRP1 [79, 80].

B-cell lymphoma-2 (Bcl-2) is an anti-apoptotic factor. From the perspective of apoptosis, the inhibition of apoptosis and the abnormal accumulation of cells are common characteristics of tumors, and studies have shown that the expression of Bcl-2 is increased in various malignant tumors [81]. The investigation of Bcl-2 in adenomyosis revealed that the expression of Bcl-2 in the endometria and stromal cells of adenomyosis patients was significantly greater than that in normal control patients, which enhanced the antiapoptotic ability of endometrial cells and reduced their sensitivity to apoptosis; consequently, the cells escaped apoptosis, which resulted in the implantation of ectopic sites, thereby eventually leading to the formation of ectopic lesions [81].

#### Estrogen enhances myometrial smooth muscle contraction, thus leading to the aggravation of adenomyosis

**Fig. 3:**
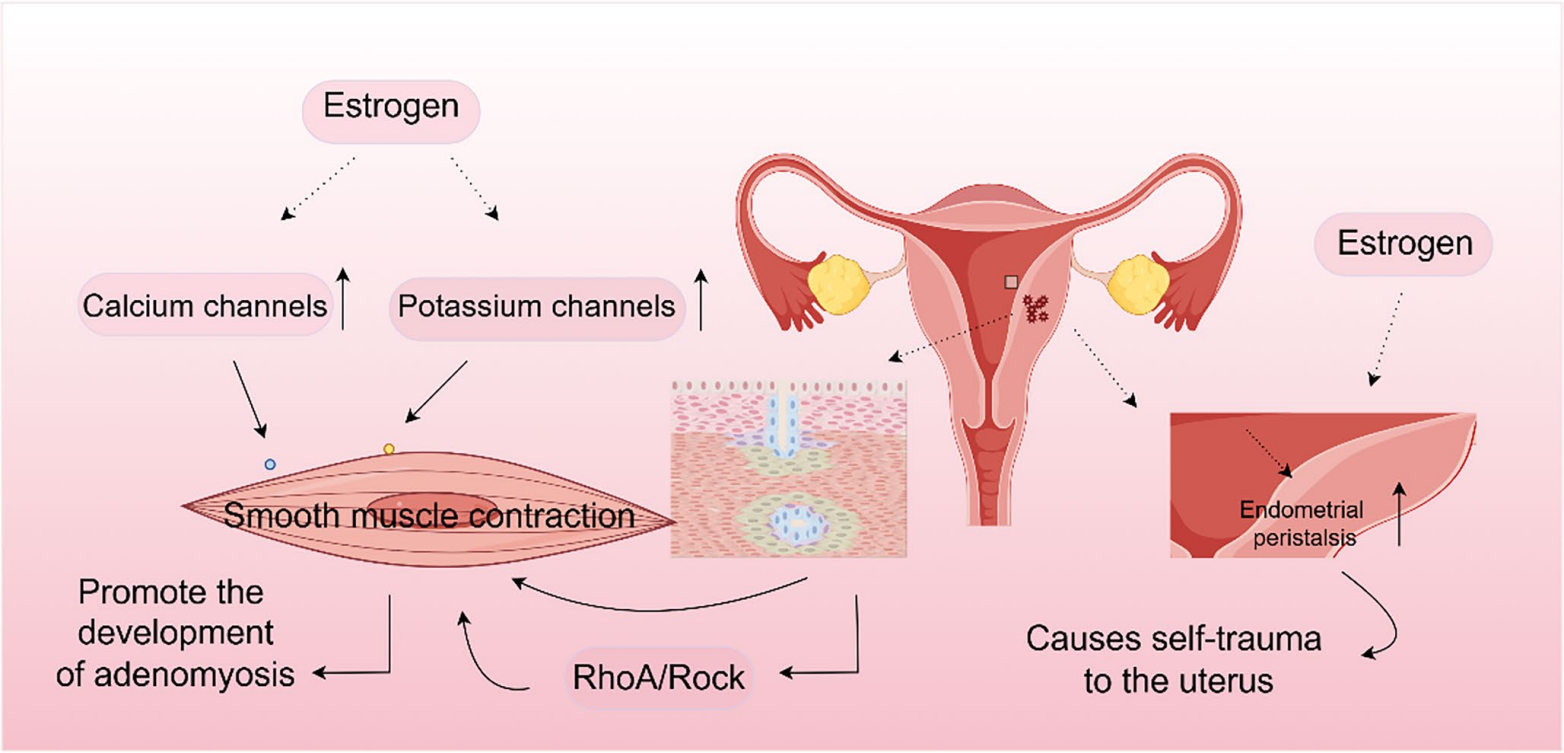
Estrogen promotes the development of adenomyosis by stimulating uterine smooth muscle contraction. Estrogen stimulates uterine peristalsis by up-regulating the expression of oxytocin receptor, resulting in obvious uterine trauma. Estrogen can also promote the contraction of uterine smooth muscle by affecting ion channels and RhoA/Rock signaling pathway, thereby exacerbating adenomyosis

The increase in local estrogen in adenomyosis can upregulate the expression of the oxytocin receptor, cause uterine hyperactivity and stimulate uterine peristalsis. Excessive peristalsis can lead to uterine trauma and the infiltration and growth of the basal endometrium into the uterine layer, which eventually leads to the occurrence of adenomyosis [15, 82]. Moreover, oxytocin, which is mediated by the oxytocin receptor, is involved in the release of prostaglandin F2α (PGF2α) from endometrial cells. The increased production of PGF2α promotes the production of prostaglandins and upregulates COX-2, which correspondingly promotes the production of estrogen [83–88]. (Fig. 3)

The development of adenomyosis caused by estrogen may be related to changes in ion channels. For example, excessive estrogen can lead to the abnormal expression of potassium channels in the uterine smooth muscle cells of adenomyosis patients and may lead to progesterone resistance [89]. Similarly, estrogen can induce an increase in calcium ions in smooth muscle cells at the endometrial-myometrial interface, thus resulting in abnormal muscle contraction at the endometrial-myometrial interface, which is also related to the pathogenesis of adenomyosis [90–92].

The RhoA/ROCK signaling pathway has been investigated in many tumors, and it is believed that this pathway can promote the invasion of tumor cells. Many studies have confirmed that the RhoA/ROCK signaling pathway can promote the invasion and growth of adenomyosis and is widely involved in the pathophysiological process of adenomyosis [93–95]. The role of the RhoA/ROCK signaling pathway is closely related to estrogen. Specifically, estrogen can enhance RhoA/ROCK-1 signal transduction to affect smooth muscle contraction in the junction area of adenomyotic lesions, and excessive proliferation of uterine smooth muscle cells aggravates the process of adenomyosis [96, 97].

The abovementioned findings demonstrate that estrogen plays an important role in the occurrence and development of adenomyosis; however, with respect to clinical medication, the effect of the simple use of antiestrogen drugs is not ideal, which indicates that the occurrence and development of adenomyosis includes not only the pathological phenomenon of an abnormal increase in estrogen but also other important pathological phenomena.

## The occurrence of the inflammatory response—an important process in the progression of adenomyosis

The abnormal accumulation of immune cells (such as macrophages and lymphocytes) in the ectopic endometria of adenomyosis patients suggests that there is an inflammatory response that occurs in the ectopic endometrium [98, 99]. A previous study demonstrated that the infiltration of macrophages in the endometria of adenomyosis patients was significantly greater than that in the endometria of normal controls; moreover, the damage to microvilli and abnormalities in microtubules of the endometria in adenomyosis patients were also significantly greater than those in the normal endometria of control patients, which is closely related to infertility [100, 101].

Inflammation not only affects immunity and endocrine function but also plays a key role in decidual formation [102]. Persistent inflammation may be the cause of spontaneous decidual dysfunction [103]. In addition, inflammation is also a factor leading to infertility in adenomyosis [104–110]. Furthermore, inflammatory factors such as IL-6, IL-10, IL-13, and tumor necrosis factor-α (TNF-α) are significantly correlated with infertility [111, 112].

### Recruitment of inflammatory cells in adenomyosis

#### Macrophages

Propst et al. reported that the expression of granulocyte-macrophage colony-stimulating factor in the glandular epithelium of adenomyosis patients was increased, which was related to the recruitment and activation of macrophages. Some studies have also revealed that the number of CD68-positive macrophages in the epithelium and stroma of adenomyotic lesions was greater than that in the normal endometrium by immunohistochemical analysis [113, 114]. These findings indicate that macrophage activation and recruitment occur during the development of adenomyosis.

Macrophages can lead to increased invasiveness of endometrial cells. Via the coculturing of endometrial stromal cells with M2 macrophages, the expression of the gene associated with retinoid-IFN-induced mortality-19 (GRIM-19) was significantly reduced, and defects in GRIM-19 reduced the apoptosis of endometrial stromal cells and increased their proliferation and migration [115]. These findings indicate that M2 macrophages can promote the occurrence of adenomyosis by inducing the proliferation and invasion of endometrial stromal cells. Similarly, Stratopoulou et al. reported that M2 macrophages can accumulate in the endometrium; moreover, when endometrial epithelial cells and stromal cells were cocultured with M2 macrophages, they were found to be more invasive [116].

Macrophages are associated with epithelial-mesenchymal transition in adenomyosis. Macrophages play a key role in the regulation of epithelial-mesenchymal transition. For example, Min et al. successfully induced epithelial-mesenchymal transition in normal endometrial epithelial cells using THP-1-derived macrophages. The expression levels of the genes encoding TGF-β1/Smad3 and IL-6/STAT3, which are considered to be important for epithelial-mesenchymal transition, were significantly increased; moreover, it was demonstrated that THP-1-derived macrophages polarized into M2 macrophages [117, 118]. Hu et al. reported that THP-1-derived macrophages were polarized to M2 macrophages after treatment with extracellular vesicles from patients with adenomyosis. The expression levels of vimentin and N-cadherin, as well as activity of the PI3K/AKT pathway, in cocultured endometrial epithelial cells were also increased [119], thereby indicating that macrophages play a role in promoting epithelial-mesenchymal transition in adenomyosis. Moreover, the Notch signaling pathway is also a key regulator that induces epithelial-mesenchymal transition. Bourdon et al. reported that the expression level of Notch1 mRNA in a mouse model of adenomyosis was greater than that in the control group. Increases in M1 macrophages and natural killer cells induce Notch signaling, thereby promoting epithelial-mesenchymal transition and leading to the occurrence of adenomyosis [120].

#### Mast cells

Mast cells can be recruited into the microenvironment by chemokines released by tumor cells, thereby recruiting eosinophils and neutrophils and stimulating them to release proangiogenic factors and matrix metalloproteinases. These factors increase neovascularization, support tumor invasion, change the tumor microenvironment, and play a role in promoting tumors. In the ectopic lesions of adenomyosis, a significant number of mast cells were also observed. Che et al. reported that mifepristone can reduce the number of mast cells and inhibit their activation in a study of the mechanism of dysmenorrhea caused by mifepristone in the treatment of AM, thus indicating that mast cells are also involved in the progression of adenomyosis [121–124].

IL-17 secreted by mast cells in ectopic lesions of adenomyosis is abnormally enriched. IL-17 causes receptor dimerization by binding to the IL-17 receptor; moreover, it promotes JAK recruitment and phosphorylation and forms STAT docking sites. STAT tyrosine is subsequently phosphorylated and activated. The activation of the JAK-STAT signaling pathway can upregulate the expression of NOX4, thus promoting angiogenesis [125–127].

Mast cells mainly secrete a variety of mediators, such as histamine, prostaglandins and a large number of cytokines (including tumor necrosis factor and interleukin cytokines), thus causing clinical symptoms such as adenomyosis pain, as well as leading to the abnormal proliferation of adenomyosis lesions and promoting the progression of adenomyosis [128–133].

#### T cells

By comparing the expression of T-cell markers in ectopic and normal endometrial epithelial cells from adenomyosis patients, Bulmer et al. reported that the number of T cells was increased in the ectopic endometrium [134]. Among these cells, the increase in CD8 + T cells was closely related to the severity of adenomyosis, and the change in NKG2A+CD8 + T-cell subsets was the most significant observed effect. The increased expression of histocompatibility leukocyte antigen E (HLA-E) and IL-15 in the uterine microenvironment promotes the expression of NKG2A on CD8 + T cells, which leads to the depletion of CD8 + T cells and cannot prevent endometrial cells from infiltrating into the muscular layer [135].

T-cell immunoglobulin mucin molecule 3 (TIM-3) binds to galectin-9 (Gal-9) in activated CD4 + Th1 and CD8 + T cells, and its persistent expression can also lead to T-cell failure, thus resulting in T cells being in a dysfunctional state. This scenario is accompanied by the secretion of large amounts of IL-10, perforin and granzyme, which can promote the migration and invasion of cancer cells [136, 137]. Pu Huang et al. reported that the expression of TIM-3/Gal-9 was increased in ectopic and eutopic endometrial glandular epithelial cells, thereby regulating the growth, migration or invasion of ectopic endometrial cells and promoting the development of adenomyosis [138].

The ratio of Th17/regulatory T cells in the peripheral blood of patients with adenomyosis is proportional to the level of CA125 and the degree of dysmenorrhea, whereas increases in TGF-β and IL-6 levels in the plasma of patients with adenomyosis and increases in STAT3 transcription, can promote Th17 differentiation [139–143]. Macrophages and their secreted product (TGF-β) can activate Th17 cells, promote their differentiation, and increase the production of IL-17 and IL-10 to indirectly affect inflammation [144–147].

#### Natural killer cells

Although previous studies have revealed no difference in NK cell populations between women with and without adenomyosis, the expression of killer cell inhibitory receptors on endometrial NK cells in women with adenomyosis is reduced, which is presumed to be caused by the activation of NK cytotoxicity in women with adenomyosis to eliminate the compensatory effect of abnormal endometrial cells [148]. NKG2A is considered an inhibitory receptor in the natural killer cell 2 (NKG2) family and is mainly expressed on NK cells. Studies have revealed that CD8 + T-cell subsets eliciting depletions in NKG2A are associated with the severity of adenomyosis, and a significant increase in the number of these cells in the eutopic endometrium and in ectopic lesions has been reported.

As a type of lymphocyte that exhibits both T-cell and NK cell characteristics, NKT cells regulate cell differentiation. Studies have shown that IGFBP5 is highly expressed in nerve fibers labeled with neurogenic protein in slides of adenomyosis tissues and in surrounding SFRP + NKT cells; additionally, IGFBP5 can regulate the hypersensitivity responses of nociceptive neurons in a mouse model of peripheral nerve injury, and the high expression of IGFBP5 in SFRP + NKT cells leads to adenomyosis pain [149].

Previous studies have revealed changes in NK cell activity before and after treatment for adenomyosis; specifically, the results demonstrated that NK cell activity increases from 37.7 +/- 19.0% to 50.8 +/- 18.2% after treatment with gonadotropin-releasing hormone agonists [150]. Another study reported that, compared with that in untreated patients, the number of infiltrating NK cells in the eutopic endometrial glands of patients with adenomyosis was increased after one month of oral administration of dienogest treatment [151].

**Fig. 4:**
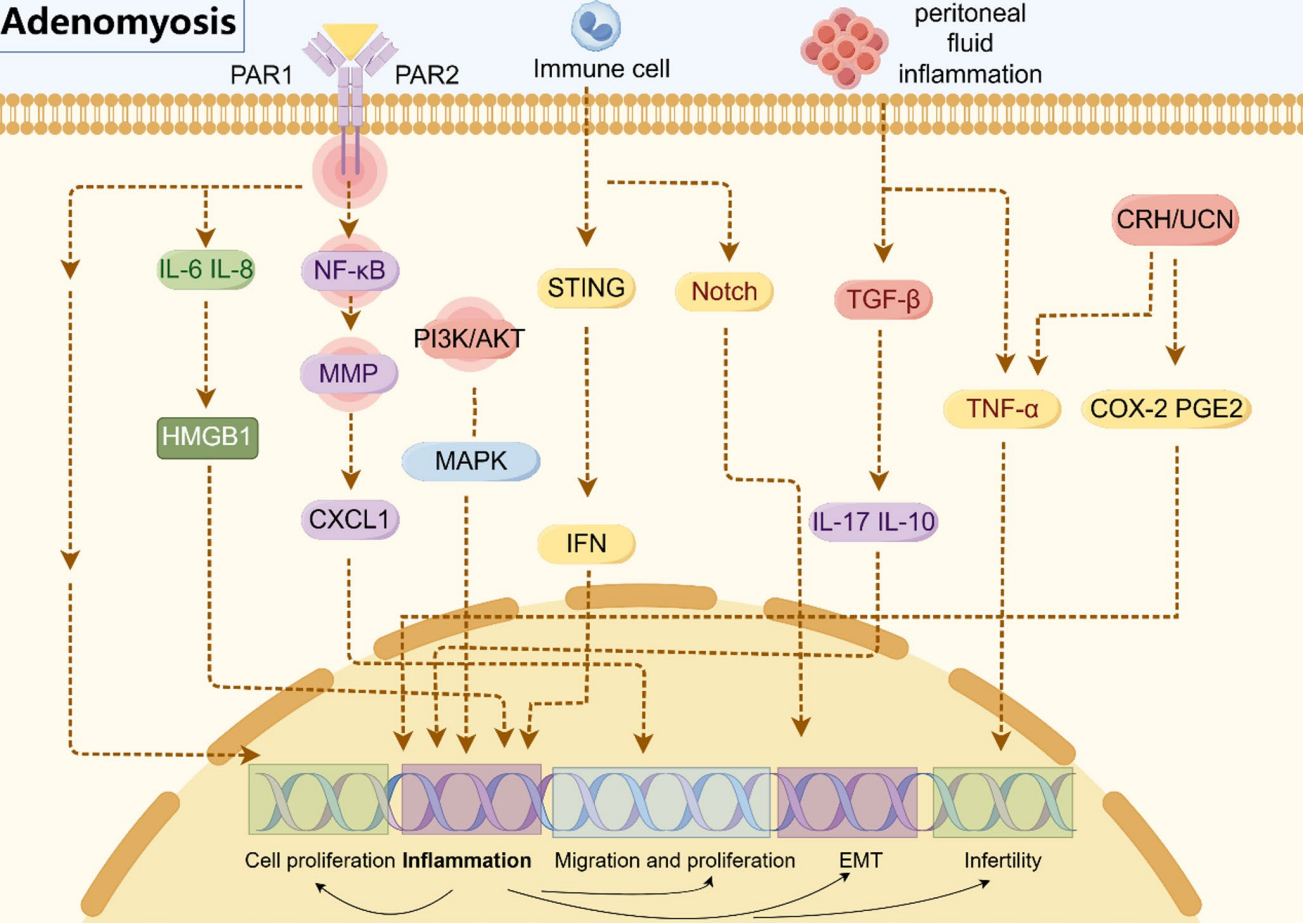
The related signaling pathways of inflammation affect the pathogenesis of adenomyosis. Inflammatory mediators such as interleukins and the NF-κB pathway promote the pathogenesis of adenomyosis, and the recruitment of inflammatory cells also leads to the invasion of endometrial epithelial cells and epithelial-mesenchymal transition, thereby promoting the pathogenesis of adenomyosis. This figure was created by Figdraw. *PAR* protease activated receptor, *HMGB1* high mobility group box 1, *NF-κB* nuclear factor kappa-B, *MMP* matrix metalloproteinase, *CXCL1* CXC motif chemokine ligand 1, *PI3K/AKT* phosphatidylinositol 3 kinase/protein kinase B, *MAPK* mitogen-activated protein kinase, *STING* stimulator of interferon genes, *IFN* interferon, *TGF-β* transforming growth factor-β, *TNF-α*, tumor necrosis factor-α, *CRH/UCN* corticotropin-releasing hormone/urinary corticotropin, *COX2* cyclooxygenase 2, *PGE2* prostaglandin E2

In adenomyosis, the increased number of activated macrophages, natural killer cells and T helper cells in the perivascular area and in the myometrium remodeling area can lead to neurogenic inflammation and increased sensitivity of nociceptors, thus eventually leading to the activation of peripheral nerve fibers and the generation of pain [152]. A number of studies have shown that the interaction between inflammation-mediated lesions and nerve fibers plays an important role in ectopic endometrial-related pain [105, 153, 154]. Therefore, the abnormal recruitment of immune cells is an important process in the development of adenomyosis and is closely related to the clinical symptoms that are caused by adenomyosis. (Fig. 4)

### Role of inflammatory mediators in adenomyosis

#### IL-6 and IL-8

IL-6 is a pleiotropic cytokine that plays an important role in immune regulation and inflammation; additionally, previous studies have shown that IL-6 plays an important role in adenomyosis-related inflammation [141, 155, 156]. In addition to promoting the expression of inflammatory pathways involving nuclear factor kappa-B (NF-κB) and matrix metalloproteinase (MMP) [157–159], IL-6 can also directly stimulate the proliferation of endometrial stromal cells [112]. The expression of IL-8 in the lesion epithelium of patients with adenomyosis is relatively high [160, 161], and it directly stimulates the proliferation of endometrial cells [162]. Moreover, IL-8, which is a marker of endometriosis, seems to function best among all of the other chemokines with respect to this effect [162, 163].

The overexpression of IL-6 and IL-8 has been shown to be positively correlated with the expression of high mobility group box 1 (HMGB1) in the endometria of adenomyosis patients. The knockdown of HMGB1 results in the inhibition of inflammatory cytokines such as IL-6, TNF-α and IL-1β [164–166]. Moreover, the silencing of HMGB-1 leads to the inhibition of inflammatory cytokines and the regulation of autophagy under hypoxic conditions [164, 165]. These findings indicate that the HMGB1-mediated inflammatory response promotes the development of adenomyosis [167].

An increase in IL-6 and IL-8 can be induced by the activation of protease-activated receptor 2 in endometrial stromal cells. Protease-activated receptor 2 can convert allergic reactions and coagulation reactions into inflammatory reactions, and the activation of protease-activated receptor 2 can stimulate the proliferation of endometrial stromal cells [168], which can promote the development of adenomyosis.

The expression of IL-6 and IL-8 is positively correlated with the expression of lipoxygenase-5 (LOX-5) and COX-2 in the endometrium. COX-2 and LOX-5 can produce an inflammatory microenvironment and lead to the development of cancer by promoting proliferation and angiogenesis. In adenomyosis, the overexpression of these factors in dysmenorrhea is associated with menorrhagia. Therefore, COX-2 and LOX-5 may promote the development of adenomyosis by regulating inflammatory pathways [57].

#### IL-1β

The NLRP3 inflammasome is also involved in the pathophysiology of adenomyosis by activating cysteine protease caspase-1 to produce active IL-1β and IL-18 [169–171]. IL-1β can induce WEE1 expression in endometrial stromal cells. WEE1 is a protein kinase involved in cell cycle regulation and the DNA damage response. WEE1 can also promote the migration and fibrosis of endometrial stromal cells via the Wnt/β-catenin signaling pathway [172].

Neuronal injury-inducing protein 1 (Ninj1) has been observed to promote neurite outgrowth. In adenomyosis, Ninj1 can be induced by inflammatory stimulation. Specifically, IL-1β can significantly increase the expression of Ninj1 mRNA in human endometrial stromal cells, which may be related to the pathogenesis of pain symptoms [173].

The high expression of IL-1β, corticotropin-releasing hormone (CRH) and urinary corticotropin (UCN) in adenomyosis nodules indicates that inflammation is significantly involved in the pathogenesis of adenomyosis [174, 175]. CRH and UCN have been shown to activate COX-2 in other tissues, and UCN significantly increases the secretion of TNF-α and IL-4. Furthermore, the high expression of CRH and UCN in adenomyosis may also lead to increased prostaglandin synthesis [30, 176], which exacerbates the occurrence of inflammation.

#### NF-κB

Increased activation of NF-κB is considered to be closely related to the pathogenesis and treatment of adenomyosis [177]. This effect stimulates inflammation and cell proliferation in endometriotic cells, inhibits apoptosis, promotes angiogenesis, and plays a key role in the pathogenesis of adenomyosis [43, 178–183]. NF-κB is an important inflammatory regulatory factor that can induce the secretion of IL-6, TNF-α and IL-1β and promote the development of adenomyosis [184]. A previous study revealed that the expression of the NF-κB p65 subunit was increased in adenomyosis nodules [185, 186]. Moreover, stromal cells from the ectopic endometrium and adenomyosis nodules have been demonstrated to exhibit increased immunoreactivity for the NF-κB p65 subunit [187].

The high migration and invasion ability of endometrial cells is considered to be the primary mechanism underlying the implantation and expansion of ectopic lesions. Previous studies have shown that endometrial epithelial cells can induce the expression and secretion of MMPs in normal endometrial stromal cells via NF-κB, thus promoting the implantation and expansion of ectopic lesions [188–190]. The NF-κB signaling pathway induces the expression of CXC motif chemokine ligand 1 (CXCL1) in human endometrial epithelial cells and participates in both the inflammatory response and angiogenesis, which also leads to the migration and invasion of vascular endothelial cells [191]. A previous study demonstrated that the knockdown of IKKβ by short interfering RNA significantly inhibited the migration and invasion of ectopic endometrial cells, thereby further demonstrating the key role of NF-κB signal transduction in ectopic endometrial implantation [192].

#### PI3K

Studies have shown that the activation of the PI3K/AKT signaling pathway can significantly increase the levels of pyroptosis and various inflammatory factors, such as IL-1β, IL-18 and TLR4; moreover, it is associated with angiogenesis, cell proliferation and migration [193, 194]. Nuclear receptor 4 A (NR4A) is a key regulator of decidualization that can promote and regulate the decidualization of endometrial stromal cells by increasing the expression of decidualization marker genes. A previous study revealed that high expression of p-AKT (Ser473) leads to a decrease in the expression of the NR4A receptor and its target gene FOXO1A, thus damaging the decidualization of stromal cells in adenomyosis, which indicates a new mechanism of impaired decidualization in patients with adenomyosis [195].

The immunohistochemical expression of the G protein-coupled estrogen receptor (GPER) in the ectopic endometria of adenomyosis patients is significantly different [196]. Studies have confirmed that GPER can be activated in a hypoxic environment, regulate the PI3K/Akt signaling pathway, further affect cell invasion, regulate the cell cycle and inhibit apoptosis. It can also promote cell migration and epithelial-mesenchymal transition by activating fibroblasts, inducing angiogenesis and promoting the development of adenomyosis [39, 197–200].

Long noncoding RNAs (lncRNAs) do not have protein-coding functions but are involved in various biological processes, including cell proliferation, invasion, migration and apoptosis at the posttranscriptional level. A previous study revealed that the expression of Linc-ROR in the ectopic endometria of adenomyosis patients was greater than that in the eutopic endometrium and normal endometrium and that the expression of Akt and p-Akt was also increased. Moreover, when Linc-ROR was knocked down in adenomyosis cells, cell proliferation was significantly reduced [201], thus indicating that Linc-ROR can promote the proliferation of endometrial cells by activating the PI3K/Akt pathway.

#### MMPs

MMPs can coordinate inflammatory functions. These molecules not only regulate the migration of inflammatory cells from the vascular system to the site of tissue inflammation but also regulate the recruitment and inflow of inflammatory cells to the site of inflammation by processing extracellular matrix components, growth factors, cytokines and chemokines [202]. It has been demonstrated that the expression of MMP-2 and MMP9 is increased in epithelial cells and stromal cells in adenomyosis [203]. Moreover, MMPs may promote the development of adenomyosis by promoting the invasion of endometrial tissue into the myometrium and angiogenesis in adenomyosis implants [204, 205]. Another study revealed that the C allele of the MMP-2–1306 C/T polymorphism significantly increased the risk of adenomyosis [206], and the expression of MMP in the endometrium was most pronounced during menstruation [207].

P21-activated kinases (Paks) belong to the serine/threonine kinase family and can regulate a variety of cell activities and cancer cell signal transduction networks. The increased expression of Pak4 can promote the development of adenomyosis by upregulating the activities of MMP-2 and MMP-9 in endometrial cells, thereby leading to increased invasiveness [208]. Yang et al. reported that the expression of MMP-9 in endometrial epithelial cells treated with estrogen was upregulated and that the migration ability of epithelial cells was also enhanced [209].

#### Other inflammatory mediators

Previous studies have shown that chemokines such as CXCL12, CCL26, CCL21, MCP-1, CXCL8 and CCL3 are upregulated in the endometria of adenomyosis patients [161, 210–214]. CCR1 is a proinflammatory chemokine receptor. Compared with that in the endometria of women without adenomyosis, the mRNA level of CCR1 in the endometria of patients with adenomyosis is significantly increased, and the protein level of CCR1 is also increased, which leads to the secretion of proinflammatory factors and promotes the development of adenomyosis [215]. The expression of CXCL12 and CXCR4 in ectopic lesions in adenomyosis is also significantly increased, and CXCL12/CXCR4 has been shown to play an important role in the proliferation and invasion of tumor cells. Moreover, these chemokines play important roles in tumor-associated angiogenesis and organ-specific tumor metastasis. Therefore, the synthesis and release of CXCL12 and CXCR4 by ectopic endometrial cells are related to the migration of endometrial cells to the myometrium [216]. In addition, the expression of CCL26 mRNA in the endometria of adenomyosis patients is significantly greater than that in the endometria of patients without adenomyosis, and CCL26 is involved in the pathogenesis of adenomyosis by inducing epithelial-mesenchymal transition in the basal layer of the endometrium [217].

The expression level of stimulator of interferon genes (STING) in the epithelial cells of adenomyosis patients is significantly increased, and STING can initiate and coordinate the host’s innate immunity by inducing type I interferon (IFN); moreover, the expression level of STING is related to the degree of epithelial lymphocyte infiltration, which indicates that chronic inflammation of the endometrium plays an important role in adenomyosis [218].

IL-37, which is a newly discovered anti-inflammatory factor, can reduce the expression of MMP2 via the Rac1/NF-κB signaling pathway and effectively inhibit the migration and invasion of endometrial cancer cells and the growth of endometriosis-like lesions [219, 220]. However, the mRNA and protein expression levels of IL-37 are low in the eutopic and ectopic endometria of adenomyosis patients [221].

The levels of proinflammatory cytokines (such as IL-6, IL-1β, and IL-8, among others), anti-inflammatory cytokines (such as IL-10 and IL-22, among others), transforming growth factor-β (TGF-β), monocyte chemoattractant protein-1, transcription factors (such as NF-κB, among others) and high-sensitivity C-reactive protein are increased in patients with adenomyosis [144, 155–229]. The expression of inflammatory mediators can affect angiogenesis, endometrial cell invasion, immune cell recruitment and activation, and epithelial-mesenchymal transition [230–236]. Therefore, in addition to the pathological phenomenon of an abnormal increase in estrogen, the occurrence of an inflammatory response is a critical factor for the occurrence and development of adenomyosis. With respect to the mechanism of tissue damage and repair, inflammation can maintain a high estrogen environment, and estrogen can aggravate the inflammatory response; thus, there are a close relationship between estrogen and the inflammatory response.

## The vicious cycle of inflammation and estrogen promotes the development of adenomyosis

**Fig. 5:**
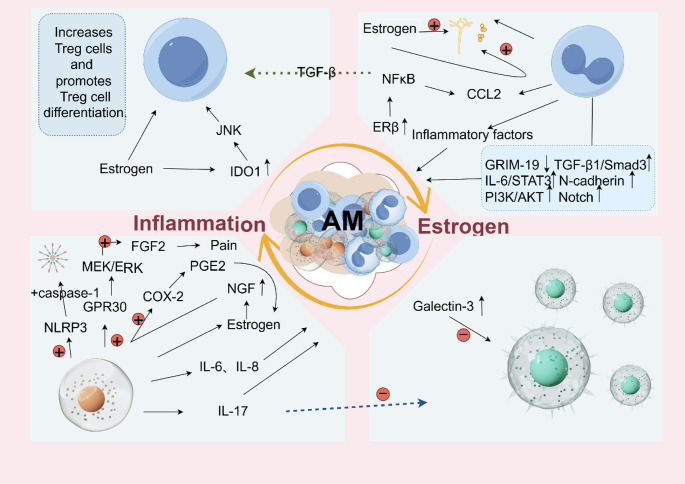
The interaction between inflammation and estrogen affects the pathogenesis of adenomyosis. Estrogen aggravates the inflammatory response by interacting with inflammatory cells, inflammatory factors and inflammatory pathways, and the inflammatory response can also maintain a high estrogen state in adenomyosis, which promotes the pathogenesis of adenomyosis. This figure was created by Figdraw. *JNK* C-Jun N-terminal kinase, *IDO1* indoleamine 2,3-dioxygenase-1, *FGF2* fibroblast growth factor 2, *MEK/ERK* mitogen-activated protein kinase kinase/extracellular signal-regulated kinase, *PGE2* prostaglandin E2, *COX2* cyclooxygenase 2, *NLRP3* NOD-like receptor hot protein domain-related protein 3, *GPR30* G protein-coupled estrogen receptor 30, *NGF* nerve growth factor, *NFκB* nuclear factor kappa-B, *ERβ* estrogen receptor β, *GRIM-19* gene associated with retinoid-IFN-induced mortality-19, *TGF-β1/Smad3* transforming growth factor-β1/small mothers against decapentaplegic, *STAT3* signal transducer and activator of transcription 3, PI3K/AKT, phosphatidylinositol 3 kinase/protein kinase B

In the widely studied mechanism of tissue damage and repair, it is believed that endometrial peristalsis causes chronic self-injury to produce an inflammatory reaction, activates IL-1, and promotes estrogen production via COX-2 and prostaglandins; additionally, an abnormal increase in estrogen can elicit hyperactivity of endometrial peristalsis and aggravate the inflammatory reaction. Therefore, an increase in estrogen can aggravate the inflammatory response in a variety of ways and that the inflammatory response can maintain the local high estrogen state in many ways. Thus, there is a complex interaction between estrogen and inflammation, which jointly promotes the occurrence and development of adenomyosis. (Fig. 5)

### Interaction between estrogen and inflammatory cells

#### Estrogen and macrophages

Estrogen can promote the secretion of chemokines in peripheral nerves and enhance the recruitment and polarization of macrophages in endometriosis. Studies have shown that the coculture of macrophages with estrogen increases the concentration of neurotrophic factor 3, which stimulates nerve growth [237]. Under the influence of estrogen, the coexistence of macrophages and nerves induces a new type of neuroimmune communication. The continuous stimulation of inflammatory cytokines from macrophages to peripheral nerve nociceptors aggravates neuroinflammation by releasing inflammatory neurotransmitters, which eventually leads to neuropathic pain [238].

In addition, previous studies have shown that the expression of estrogen receptors in the macrophages of women with endometriosis is significantly greater than that in the control group, which was demonstrated by detecting the estrogen receptors of macrophages in the peritoneal fluid of patients with endometriosis [239]. The estrogen receptor also has a recruitment effect on macrophages. Gou et al. reported that the high expression of estrogen receptor β can produce CCL2 via the signal transduction of NF-κB and recruit macrophages to the ectopic environment of the endometrium, whereas the recruited macrophages can promote the proliferation and colony formation of endometrial stromal cells [240].

CD200 is an immunomodulatory factor and a type I glycosylated protein that is located on the cell surface. Studies have shown that the CD200 protein can be induced by estrogen; moreover, with increasing CD200 concentration, the phagocytosis rate of macrophages decreases, which leads to an impaired macrophage-mediated immune response in endometriosis [241], thereby potentially leading to the development of adenomyosis.

#### Estrogen and mast cells

The number of mast cells in ectopic lesions of adenomyosis is significantly increased. Studies have shown that the levels of the proinflammatory factors IL-6 and IL-8 are increased in human endometrial stromal cells cultured in the conditioned medium of endometrial ectopic epithelial cells and human mast cells. A high estrogen environment can promote the recruitment of mast cells and aggravate the inflammatory response to promote the pathogenesis of adenomyosis [242].

Nerve growth factor (NGF) plays an important role in the generation of pain, nerve plasticity and the release of inflammatory factors. Studies have shown that NGF is significantly upregulated in tamoxifen-induced adenomyosis mice [12]. In addition, 17β-estradiol can stimulate the production of NGF in endometrial stromal cells in adenomyomas and subsequently induce the expression of cyclooxygenase in mast cells, increase the production of prostaglandin E2, and promote the proliferation of endometrial stromal cells in adenomyomas and the synthesis of aromatase [243, 244]. Moreover, it can also induce the production of TNF-α and IL-6 to further promote the development of adenomyosis [244].

Studies have shown that estrogen can promote the secretion of fibroblast growth factor 2 in endometriosis lesions via the G protein-coupled estrogen receptor 30 (GPR30) and MEK/ERK pathways in mast cells. Specifically, a previous study showed that the concentration of fibroblast growth factor 2 in the pelvic fluid of the mild pain group and severe pain group was greater than that in the pelvic fluid of patients without endometriosis-related pain, thereby indicating that the increase in fibroblast growth factor (FGF2) secretion in mast cells may be a potential cause of related pain caused by endometriosis in women [245].

Estrogen can lead to an increase in the rate of degranulation of mast cells, and the degranulation of mast cells is related to endometriosis-related dysmenorrhea, which itself is related to estrogen promoting the release of nerve growth factor. These effects consequently promote the growth of neurites in PC12 cells and upregulate the expression levels of Nav1.8 and transient receptor potential cation channels to increase dorsal root ganglion cell sensitivity [246], which may also be a related factor leading to the occurrence of pain in adenomyosis.

Estrogen promotes the activation of mast cells, and the expression of NOD-like receptor hot protein domain-related protein 3 (NLRP3) in mast cells is significantly increased after estrogen treatment. NLRP3 is an intracellular receptor that interacts with caspase-1 to form the NLRP3 inflammasome, and estrogen can strongly activate the inflammasome via potassium efflux in mast cells [247], thereby enhancing the pathogenesis of adenomyosis.

#### Estrogen and T cells

Studies have shown that estrogen can promote the expansion of Tregs and the production of cytokines such as IL-10 and TGF-β; moreover, the mRNA expression of ERα in amplified Tregs is increased. An increase in Tregs promotes the invasion and survival of endometrial stromal cells [248]. In addition, indoleamine 2,3-dioxygenase-1 (IDO1) is highly expressed in ectopic endometrial stromal cells. This molecule is a rate-limiting enzyme that catalyzes the synthesis of tryptophan and can promote the survival, proliferation and invasion of endometrial stromal cells via the c-Jun N-terminal kinase (JNK) signaling pathway [249] Estrogen can upregulate the expression of IDO1 and participate in the differentiation of Tregs, thus affecting the ability of the immune system to eliminate ectopic endometrial tissue [250].

#### Estrogen and natural killer cells

The increased expression of galectin-3 can be induced by estrogen, and studies have shown that galectin-3 can impair the toxicity of natural killer cells in the peritoneal cavity to endometrial stromal cells, enhance the peritoneal implantation of endometrial stromal cells, and ultimately promote the adhesion and migration of endometrial stromal cells to the lesion [251, 252], which promotes the occurrence of adenomyosis. Similarly, studies have shown that galectin-1, −3 and − 9 are overexpressed in the endometria of women with endometriosis and are related to the development of endometriosis-related tumors [253, 254]. Therefore, the development of adenomyosis cannot be ignored with respect to this factor.

IL-17 A is a proinflammatory cytokine that is closely related to the estrogen-COX-2 axis. IL-17 A has been found to promote the survival of endometrial cells by upregulating antiapoptotic genes, and IL-17 A can also inhibit natural killer cell-mediated cytotoxicity and promote angiogenesis and the proinflammatory environment in the peritoneal cavity [255, 256]. IL-17 A also enhances the expression of COX-2 mRNA and plays a role in the development of endometriosis by promoting the proliferation of ectopic stromal cells [257]. In addition, elevated estrogen levels promote the production of IL-15 in the ectopic endometrium, thereby stimulating the growth and invasion of endometrial stem cells and inhibiting the cytotoxic activity of natural killer cells [258, 259].

The Toll-like receptor 4 (TLR4) signaling pathway is essential for the pathogenesis of adenomyosis [260]. TLR4 signals that are activated by endogenous ligands promote the secretion of various cytokines and growth factors, stimulate the proliferation of endometrial cells, and recruit and activate immune cells (such as macrophages, DCs, and NK cells), estrogen is also involved in promoting the activation of TLR4, the proliferation and invasion of stromal cells further induce and amplify the local inflammatory response, thus ultimately leading to the development of adenomyosis [261–264].

### Interactions between estrogen and inflammatory mediators

#### Estrogen and interleukins

The mechanism of tissue damage and repair in adenomyosis is physiologically associated with the local production of IL-1. IL-1 activates COX-2 and induces the production of prostaglandin E2; subsequently, steroids produce acute regulatory proteins, aromatase is activated, and testosterone is aromatized into estradiol, which helps in maintaining local estrogen overproduction [15, 58, 59, 265–268]. Prostaglandin E2 also promotes angiogenesis through its effects on estrogen and the upregulation of vascular endothelial growth factor [269]. Elevated levels of prostaglandins in the peritoneal fluid and the expression of aromatase in the endometrium are considered to exert specific impacts on endometriosis-associated infertility [267, 270]. In addition, the combination of IL-4 and prostaglandin E2 enhances estrogen production in the ectopic endometrium [271].

Hyperestrogenemia has also been shown to stimulate the production of IL-10, which is a cytokine exhibiting an immunosuppressive ability. IL-10 is highly expressed in the eutopic and ectopic endometria of women with adenomyosis. This observation may explain the persistence of ectopic lesions in the myometrium without the elimination of the host immune system [183].

#### Estrogen and inflammatory pathways

Studies have shown that estrogen can increase the activity of NF-κB in ectopic endometrial cells. Estradiol can activate the PI3K/AKt pathway via the NF-κB/PTEN-dependent pathway, thereby promoting the proliferation of ectopic endometrial cells [272]. Estradiol has proinflammatory and antiapoptotic effects on endometrial cells. Moreover, estradiol can induce inflammatory responses mediated by local chemokines at normal physiological concentrations and enhance cell survival mechanisms mediated by extracellular signal-regulated kinases and Bcl-2. These effects seem to be exacerbated in women with endometriosis [273]. Furthermore, platelets have also been demonstrated to upregulate aromatase by activating the NF-κB and/or TGF-β1 pathways, thereby increasing estrogen production [274]. In addition, estrogen can promote the release of inflammatory factors by inducing the expression of GATA-3, thereby promoting the development of endometriosis [275].

In the investigation of the mechanism of adenomyosis, we observed that the occurrence of estrogen abnormalities and inflammation is closely related, and a variety of evidence shows that there is an interaction between estrogen and inflammation, which is of far-reaching significance for further guiding clinical medication use.

## Anti-inflammatory and estrogen therapy in adenomyosis

Adenomyosis is a chronic, hormone-related disease characterized by the presence of endometrial glands and stroma within the myometrium. The most common symptoms include chronic pelvic pain, dysmenorrhea, menorrhagia, and postmenopausal bleeding, but these symptoms are not specific and cannot be used for clinical diagnosis. With the advancement of diagnostic techniques, transvaginal ultrasound and magnetic resonance imaging can help in the early diagnosis of adenomyosis and distinguish it from other gynecological diseases. Based on these two examination methods, it can be classified into: diffuse adenomyosis; focal adenomyosis; and cystic adenomyosis. Early diagnosis is crucial for individualized treatment management, and drug therapy is the preferred treatment option. Selecting appropriate drugs based on the patient’s pathogenesis for intervention is extremely important [19, 276]. Currently, abnormal estrogen levels, the occurrence of inflammatory responses, and their interaction play a prominent role in the pathogenesis of adenomyosis. Therefore, we have summarized the currently applied treatment drugs targeting abnormal estrogen levels and inflammatory responses and analyzed the existing two-drug combination treatment methods.

### Estrogen-targeted therapy in adenomyosis

#### Gonadotropin-releasing hormone agonist (GnRH-as)

Gonadotropin-releasing hormone (GnRH) is a hormone released by the hypothalamus that regulates reproductive function by stimulating the pituitary gland to release follicle-stimulating hormone (FSH) and luteinizing hormone (LH). The continuous administration of GnRH-as has been shown to initially cause a ‘flare-up’ effect, after which this administration inhibited the secretion of FSH and LH, thereby resulting in the production of ovarian-blocking steroids. Therefore, GnRH-as can improve symptoms by inducing a low estrogen status and regression of endometriosis implants [277]. Studies using GnRH-as to treat endometriosis have shown that these agonists can maintain the bone mineral density of the lumbar spine, reduce angiogenesis, relieve pain, and reduce subjective side effects [278–280].

#### Gonadotropin-releasing hormone antagonists (GnRH-ants)

GnRH-ants act by blocking the GnRH receptor in the pituitary gland and immediately inhibit reproductive function by inhibiting the secretion of FSH and LH by the pituitary gland. At present, elagolix is an oral, nonpeptide gonadotropin-releasing hormone antagonist that is considered for the treatment of uterine leiomyoma complicated with adenomyosis. This drug type has a dose-dependent and fast reversible effect on the pituitary-gonadal axis. Moreover, it can effectively improve dysmenorrhea and nonmenstrual pelvic pain, reduce excessive menstrual volume and improve the quality of life of patients [281, 282]. In addition, linzagolix is a new type of GnRH-ant that is being investigated for the treatment of adenomyosis and endometriosis. Linzagolix significantly reduces the patient’s pain and excessive menstrual flow, reduces the size of the lesion, improves the patient’s quality of life, and demonstrates good safety [31, 283–285].

#### Aromatase inhibitors

Aromatase inhibitors specifically target aromatase. Aromatase functions by converting androgen into estrogen. In women with endometriosis, adenomyosis or leiomyoma, abnormal aromatase expression is stimulated by prostaglandin E2. This phenomenon appears to lead to increased estrogen production and further expression of prostaglandin E2, thereby leading to inflammation in ectopic endometriosis implants [265]. The selective inhibition of aromatase via drugs such as anastrozole or letrozole (used either alone or in combination with GnRH-as) has shown good efficacy in adenomyosis and endometriosis [286–289]. Badawy et al. randomly assigned patients to receive oral letrozole (2.5 mg/day) or subcutaneous injections of GnRH-as (goserelin, 3.6 mg) for 12 weeks and reported that aromatase inhibitors are as effective as GnRH agonists in reducing the volume of uterine adenomyoma and improving symptoms [286]. Kimura et al. used the aromatase inhibitor anastrozole combined with GnRH-as to treat severe adenomyosis in premenopausal women. After 8 weeks of treatment, the reduction rate in uterine volume was observed to be 60% based on magnetic resonance imaging and ultrasonography [287]. Machado et al. reported that clotrimazole interferes with the estrogen production pathway by downregulating aromatase, thereby reducing serum estrogen levels, which indicates that clotrimazole can reduce the size of endometriosis lesions, thus decreasing disease progression [290].

#### Dienogest

Progesterone exhibits a wide range of contraceptive effects. In addition, due to the fact that progesterone can inhibit the secretion of FSH and LH and ultimately inhibit the formation of ovarian steroids and alleviate the excessive estrogen production associated with adenomyosis, this hormone is also used for the treatment of adenomyosis [291]. Dienogest is an oral progesterone drug. Hirata et al. evaluated 15 patients who were treated with dienogest for 24 weeks and reported that dienogest effectively alleviated the pain of adenomyosis patients and had an inhibitory effect on estradiol; however, 5 patients experienced anemia symptoms due to uterine bleeding [292]. Uterine bleeding is the most common side effect of this treatment, according to clinical trials of the long-term use of dienogest [293, 294].

#### Ulipristal acetate

Ulipristal acetate (UPA) is a selective progesterone receptor modulator that delays ovulation and endometrial maturation by reducing serum estradiol levels [295]. This modulator has been used to treat adenomyosis symptoms; however, only limited clinical evidence supports its use. Gracia et al. reported that UPA significantly reduced bleeding and pain caused by adenomyosis through the performance of clinical trials and achieved a high amenorrhea rate in women with uterine fibroids. UPA may be a good alternative therapy for treating adenomyosis [296]. After 12 weeks of treatment with 10 mg UPA every day for patients with adenomyosis, Capmas et al. reported that UPA exhibited positive effects on abnormal uterine bleeding caused by adenomyosis, thereby reducing the pain of patients and improving their quality of life [297]. (Table 1)

**Table 1:** Medications for estrogen therapy

Drugs	Category	Effects	References
Leuprolide acetate	Gonadotrophin-releasing hormone agonist	A hypo-estrogenic effect	Khaleque Newaz Khan (2009)
Letrozole	Aromatase inhibitor	Reduce adenomyoma volume and improving symptoms	Ahmed M Badawy (2012)
Anastrozole	Aromatase inhibitor	Reduce uterine volume	Fuminori Kimura (2007)
Dienogest	Progestin	Reduce adenomyosis-associated pelvic pain、 CA-125 and CA19-9 levels; A modest Suppression of estradiol	Tetsuya Hirata (2014)
Ulipristal acetate	A selective progesterone receptor modulator	A significant reduction in the clinical symptoms of adenomyosis (bleeding and pain)	Meritxell Gracia (2018)

### Targeting inflammation in adenomyosis

#### Nonsteroidal anti-inflammatory drugs (NSAIDs)

Menstrual pain is one of the common clinical symptoms of adenomyosis, and nonsteroidal anti-inflammatory drugs are the most commonly used drugs for the treatment of dysmenorrhea. Their efficacy is acceptable; however, they are prone to adverse side effects when widely used. Nonsteroidal anti-inflammatory drugs can block cyclooxygenase, inhibit prostaglandins, and relieve excessive uterine contraction; therefore, they can relieve menstrual pain [291, 298]. Regarding the treatment of excessive menstrual flow, a systematic review revealed that nonsteroidal anti-inflammatory drugs can reduce the excessive menstrual flow of patients; however, the therapeutic effect of NSAIDs is not as good as that of tranexamic acid, danazol and other drugs [33].

#### Mifepristone

Mifepristone is one of the most widely used selective progesterone receptor modulators (SPRMs). It has become a long-term treatment for adenomyosis because of its various advantages, including fewer adverse reactions, good tolerance and low price [299]. Studies have shown that the addition of mifepristone to the endometrium exposed to long-acting medroxyprogesterone acetate can significantly reduce the number of mast tryptase-positive cells and that mifepristone is related to the inhibition of mast cell activity [300]. Che et al. reported that mifepristone can reduce the secretion of IL-6 and TNF-α by endometrial epithelial cells and stromal cells, limit the infiltration and degranulation of mast cells in the endometrium and ectopic endometrium, and reduce the density of nerve fibers by inhibiting the migration ability of nerve cells in adenomyosis. These findings indicate that mifepristone exhibits anti-inflammatory activity in the treatment of adenomyosis and that mifepristone can improve dysmenorrhea symptoms in patients [123, 301]. In addition, mifepristone can increase the expression of caspase 3 in the ectopic endometrium and initiate apoptosis, thus inhibiting the development of adenomyosis [302].

#### Combined oral contraceptives

Combined oral contraceptives (COCs) block follicular development and endometrial proliferation by inhibiting FSH and LH. Therefore, COCs can be used to treat the symptoms of adenomyosis [303]. In addition, COCs can also exert anti-inflammatory effects by inhibiting the expression of cyclooxygenase-2 in adenomyosis [304]. Shaaban et al. used COCs to treat 62 patients with adenomyosis for 6 months and reported that COCs reduced pain and menstrual bleeding associated with adenomyosis; however, the effect was slightly weaker than that of levonorgestrel treatment [305]. Hassanin et al. reported that COCs can effectively treat pain, bleeding and other related symptoms in patients with adenomyosis. Compared with dienogest, the therapeutic effect of dienogest is better than that of COCs; however, its side effects are greater [306].

#### Other drugs

Li et al. reported that Sanjie Zhentong capsules can treat adenomyosis by inhibiting IL-6 and IL-10 to exert anti-inflammatory effects, and the therapeutic mechanism of these capsules involves the inflammatory response, hormone regulation, cell adhesion, proliferation and angiogenesis [307]. Intravenous injections of dexamethasone can effectively reduce inflammation and pain within 24 h after uterine artery embolization in patients with adenomyosis [308]. Additionally, Feng et al. reported that the Rhein protein can dose-dependently weaken the proliferative and hypertrophic myometrium and improve adenomyosis, which is achieved by inhibiting p-p65, p-AKT and active Rac1. Moreover, in vitro experiments have revealed that the Rhein protein has a negative regulatory effect on β-catenin in stromal cells and that stimulation with IL-1β can significantly increase the nuclear translocation of β-catenin and increase the expression of its target genes. However, Rhein has an inhibitory effect on β-catenin; thus, Rhein may improve adenomyosis via anti-inflammatory pathways [184]. Animal experiments have demonstrated that berberine downregulates the expression of TRPV1, COX-2, VEGF and other pain-related genes in mice with adenomyosis. Berberine can inhibit inflammation by inhibiting the activation of NF-κB; moreover, it can reduce hyperalgesia and exert analgesic and therapeutic effects on mice with adenomyosis [309].

The abovementioned drugs that are used for inflammation or estrogen treatment can alleviate adenomyosis; however, there are deficiencies with respect to these drugs in clinical practice, and these drugs cannot achieve the ideal therapeutic effect. Therefore, the combination of estrogen inhibition and anti-inflammatory methods should be considered in the treatment of adenomyosis to achieve better therapeutic effects than the use of a single treatment. (Table 2)

**Table 2:** Drugs for the treatment of inflammation

Drugs	Category	Effects	References
Non-steroidal anti-inflammatory drugs	Non-steroidal anti-inflammatory drugs	Blocks cox-2 and inhibits PGE2	Stella Iacovides(2015)
Mifepristone	Selective progesterone receptor modulators	Anti-inflammatory; Improves menstrual cramps	Xuan Che (2020)
Combined oral contraceptives	Steroid hormone	Blocks cox-2; Anti-inflammatory	Hugo Maia Jr (2013)
Sanjie Zhentong capsule	Proprietary chinese medicines	Anti-inflammatory	Li Du (2020)

The current management of adenomyosis mainly focuses on drug intervention, but some studies have shown that appropriate lifestyle intervention can also alleviate the pain symptoms of women with adenomyosis caused by inflammation due to tissue damage. Research has shown that after 3 or 6 months of a Mediterranean diet intervention with increased fruit and vegetable intake, the levels of substances such as vitamins, folic acid, and zinc in the body increase, which directly or indirectly inhibit the occurrence of inflammatory responses and relieve pain [310, 311].

### Dual anti-estrogen and anti-inflammatory inhibition therapy

The prevalence of adenomyosis in patients with endometriosis and the prevalence of endometriosis in patients with adenomyosis are approximately 80%, and estrogen is essential for the establishment and growth of the ectopic endometrium. Endometriosis and adenomyosis are both estrogen-dependent diseases. Moreover, endometriosis and adenomyosis are often accompanied by related inflammatory processes. In the clinical treatment of endometriosis, it has been treated by combination therapy. Xue et al. reported that the total effective rate of mifepristone combined with gestrinone in the treatment of endometriosis was 90.7%, which was significantly higher than the 77.3% reported for gestrinone alone; follow-up also revealed a higher pregnancy rate in the combined drug group(Xue et al., 2016). For the treatment of pain caused by endometriosis, the combination of aromatase inhibitors with compound oral contraceptives has also yielded good results. For example, Zhao et al. treated the pain symptoms of endometriosis by using letrozole combined with oral contraceptives and reported that the pain intensity of patients was significantly reduced after combined treatment(Zhao et al., 2021). Similarly, Amsterdam et al. used a combination of anastrozole and oral contraceptives to treat 15 patients with refractory endometriosis. Among them, 14 patients experienced significant pain relief, and no adverse reactions occurred(Amsterdam et al., 2005). These findings indicate that the combination of antiestrogen and anti-inflammatory agents has potential clinical value in the treatment of endometriosis and is worthy of further study.

Similarly, in adenomyosis, patients undergoing frozen-thawed embryo transfer were pretreated with GnRH agonists and hormone replacement therapy, whereas Liu et al. used anti-inflammatory TNF-α inhibitors for peri-implantation treatment. The implantation rate and pregnancy rate of the treatment group were significantly greater than those of the untreated group(Liu et al., 2025). This finding shows that the combined application also has potential benefits in adenomyosis. Similarly, Zhu et al. evaluated the efficacy of high-intensity focused ultrasound ablation combined with mifepristone and the levonorgestrel-releasing intrauterine system in the treatment of adenomyosis and reported that the combined treatment can reduce uterine volume, effectively improve symptoms, and reduce serum CA125 levels. The efficacy of combined application is better than that of high-intensity focused ultrasound ablation alone, high-intensity focused ultrasound ablation combined with mifepristone and high-intensity focused ultrasound combined with the levonorgestrel-releasing intrauterine system(Zhu et al., 2023). These findings show that the combined application of antiestrogen and anti-inflammatory agents has positive effects on adenomyosis patients undergoing high-intensity focused ultrasound ablation.

Therefore, in the treatment of adenomyosis, the combination of antiestrogen drugs and anti-inflammatory drugs has certain advantages and application potential. Anti-inflammatory drugs can relieve the pain symptoms caused by adenomyosis and improve the quality of life of patients by inhibiting the release of inflammatory factors, reducing the local inflammatory response of the lesion, and promoting the proliferation of endometrial cells. Antiestrogen drugs reduce the excessive growth and invasion of endometrial stromal cells and vascular proliferation by inhibiting estradiol. The synergistic effect of the two can not only cut through the two key pathological pathways of inflammation and hormones and block the progression of the disease in multiple dimensions but also control the development of the disease while alleviating symptoms. Compared with single drug treatment, combination therapy can significantly improve the therapeutic effect and provide a more optimized and comprehensive treatment strategy for patients with adenomyosis. It has the potential to become an important development direction for the clinical treatment of this disease in the future.

## Discussion

Adenomyosis is an estrogen-dependent disease, and abnormalities in estrogen are highly important for its pathogenesis. Estradiol can enhance the excessive growth and invasion of endometrial stromal cells and vascular proliferation by activating various protein pathways, such as the glucocorticoid-regulated kinase 1, transmembrane glycoprotein neuropilin 1, annexin A2 and Slug-VEGF axes. It can also cause abnormal muscle contraction at the endometrial-myometrial interface via ion channels. Aromatase P-450 can aromatize local androgens in the circulation, thereby promoting estrogen biosynthesis and increasing estrogen bioavailability, which is highly important for the pathogenesis of adenomyosis. The occurrence of inflammation is also highly important for the pathogenesis of adenomyosis. The endometria of patients with adenomyosis exhibit an abnormal accumulation of immune cells, and patients with endometriosis demonstrate changes in the peritoneal inflammatory environment. Proinflammatory cytokines such as IL-6, IL-1β and IL-8 are increased in patients with adenomyosis. The occurrence of inflammation can affect the angiogenesis, invasion and migration of endometrial cells. The overexpression of HMGB1, protease-activated receptor 1, protease-activated receptor 2, lipoxygenase-5 and cyclooxygenase-2 in the endometrium is positively correlated with various inflammatory factors, such as IL-6 and IL-8, which are related to cell proliferation and dysmenorrhea in adenomyosis. Moreover, the expression of inflammatory signaling pathway components, such as NF-κB p65 and PI3K/AKT, leads to increased levels of inflammatory factors and cell proliferation, as well as the inhibition of apoptosis. It also plays an important role in adenomyosis.

Therefore, the incidence of adenomyosis is closely related to the occurrence of estrogen abnormalities and inflammation, and a variety of evidence demonstrates that the interaction between estrogen and inflammation can also affect the incidence of adenomyosis. Estrogen can promote the secretion of chemokines by peripheral nerves and enhance the recruitment and polarization of macrophages in ectopic endometrial tissues, which ultimately leads to pain. Furthermore, estrogen plays a regulatory role in the recruitment, proliferation, differentiation and function of uterine natural killer cells, thereby enhancing the peritoneal implantation of endometrial stromal cells. The expression of inflammatory factors such as IL-17 A and MMP is also related to estrogen, which leads to the migration and proliferation of endometrial epithelial cells. Estrogen can also activate the expression of inflammatory pathways such as the NF-κB and PI3K/Akt pathways in ectopic endometrial cells, thereby promoting the proliferation of ectopic endometrial cells.

The interaction between estrogen and the inflammatory response has practical importance for the treatment of adenomyosis. The combined treatment strategy of anti-inflammatory drugs and antiestrogen drugs shows the synergistic advantages of improving symptoms and controlling lesion development by targeting the dual pathological mechanism of the inflammatory response and hormone dependence of adenomyosis and provides a more potential multidimensional intervention plan for clinical treatment. Although the combination of antiestrogen drugs and anti-inflammatory drugs may have been widely used to treat adenomyosis in clinical practice, the mechanism of their combined action is not yet clear, and research attention is insufficient. Therefore, more high-quality clinical trials with large samples and long-term follow-up are still needed to clarify the optimal dose, course of treatment and individualized applicable population of different drug combinations. Moreover, the molecular biological mechanism of drug synergy should be further explored, and the potential risks and drug resistance associated with long-term treatment should be analyzed. In the future, with the deepening of the concept of precision medicine and breakthroughs in pathogenesis research, the combined application of anti-inflammatory drugs and antiestrogen drugs may become an important development direction for the clinical treatment of adenomyosis.

## Data Availability

No datasets were generated or analysed during the current study.

## References

[1] Naftalin J, Hoo W, Pateman K, Mavrelos D, Holland T, Jurkovic D (2012) How common is adenomyosis? A prospective study of prevalence using transvaginal ultrasound in a gynaecology clinic. Hum Reprod 27:3432–3439 23001775 10.1093/humrep/des332

[2] Naftalin J, Hoo W, Nunes N, Holland T, Mavrelos D, Jurkovic D (2016) Association between ultrasound features of adenomyosis and severity of menstrual pain. Ultrasound Obstet Gynecol 47:779–783 26499878 10.1002/uog.15798

[3] Pinzauti S, Lazzeri L, Tosti C, Centini G, Orlandini C, Luisi S, Zupi E, Exacoustos C, Petraglia F (2015) Transvaginal sonographic features of diffuse adenomyosis in 18-30-year-old nulligravid women without endometriosis: Association with symptoms. Ultrasound Obstet Gynecol 46:730–736 25728241 10.1002/uog.14834

[4] Upson K, Missmer SA (2020) Epidemiology of adenomyosis. Semin Reprod Med 38:89–107 33105509 10.1055/s-0040-1718920 PMC7927213

[5] Kho KA, Chen JS, Halvorson LM (2021) Diagnosis, evaluation, and treatment of adenomyosis. JAMA 326:177–178 34255015 10.1001/jama.2020.26436

[6] Vercellini P, Viganò P, Bandini V, Buggio L, Berlanda N, Somigliana E (2023) Association of endometriosis and adenomyosis with pregnancy and infertility. Fertil Steril 119:727–740 36948440 10.1016/j.fertnstert.2023.03.018

[7] Etrusco A, Barra F, Chiantera V, Ferrero S, Bogliolo S, Evangelisti G, Oral E, Pastore M, Izzotti A, Venezia R, Ceccaroni M, Laganà AS (2023) Current medical therapy for adenomyosis: From bench to bedside. Drugs 83:1595–1611 37837497 10.1007/s40265-023-01957-7 PMC10693526

[8] Bulun SE, Yildiz S, Adli M, Wei JJ (2021) Adenomyosis pathogenesis: Insights from next-generation sequencing. Hum Reprod Update 27:1086–1097 34131719 10.1093/humupd/dmab017 PMC8543024

[9] Matalliotakis IM, Kourtis AI, Panidis DK (2003) Adenomyosis. Obstet Gynecol Clin North Am 30:63–82 12699258 10.1016/s0889-8545(02)00053-0

[10] Benagiano G, Brosens I (2012) The endometrium in adenomyosis. Womens Health (Lond) 8:301–312 22554177 10.2217/whe.12.8

[11] Bergeron C, Amant F, Ferenczy A (2006) Pathology and physiopathology of adenomyosis. Best Pract Res Clin Obstet Gynaecol 20:511–21 16563870 10.1016/j.bpobgyn.2006.01.016

[12] Parrott E, Butterworth M, Green A, White IN, Greaves P (2001) Adenomyosis–a result of disordered stromal differentiation. Am J Pathol 159:623–630 11485920 10.1016/S0002-9440(10)61733-6 PMC1850567

[13] García-Solares J, Donnez J, Donnez O, Dolmans MM (2018) Pathogenesis of uterine adenomyosis: invagination or metaplasia? Fertil Steril 109:371–379 29566849 10.1016/j.fertnstert.2017.12.030

[14] Chapron C, Vannuccini S, Santulli P, Abrão MS, Carmona F, Fraser IS, Gordts S, Guo SW, Just PA, Noël JC, Pistofidis G, Van den Bosch T, Petraglia F (2020) Diagnosing adenomyosis: an integrated clinical and imaging approach. Hum Reprod Update 26:392–411 32097456 10.1093/humupd/dmz049

[15] Leyendecker G, Wildt L, Mall G (2009) The pathophysiology of endometriosis and adenomyosis: tissue injury and repair. Arch Gynecol Obstet 280:529–38 19644696 10.1007/s00404-009-1191-0 PMC2730449

[16] Shaked S, Jaffa AJ, Grisaru D, Elad D (2015) Uterine peristalsis-induced stresses within the uterine wall may sprout adenomyosis. Biomech Model Mechanobiol 14:437–444 25217062 10.1007/s10237-014-0614-4

[17] Gargett CE, Schwab KE, Deane JA (2016) Endometrial stem/progenitor cells: the first 10 years. Hum Reprod Update 22:137–163 26552890 10.1093/humupd/dmv051 PMC4755439

[18] Gargett CE (2007) Uterine stem cells: what is the evidence? Hum Reprod Update 13:87–101 16960017 10.1093/humupd/dml045

[19] Martire FG, Costantini E, D’Abate C, Schettini G, Sorrenti G, Centini G, Zupi E, Lazzeri L (2025) Endometriosis and Adenomyosis: From Pathogenesis to Follow-Up. Curr Issues Mol Biol 47 10.3390/cimb47050298 PMC12110143 40699697

[20] Ueki K, Kumagai K, Yamashita H, Li ZL, Ueki M, Otsuki Y (2004) Expression of apoptosis-related proteins in adenomyotic uteri treated with danazol and GnRH agonists. Int J Gynecol Pathol 23:248–258 15213601 10.1097/01.pgp.0000130109.80359.57

[21] Gaetje R, Kotzian S, Herrmann G, Baumann R, Starzinski-Powitz A (1995) Invasiveness of endometriotic cells in vitro. Lancet 346:1463–4 7490993 10.1016/s0140-6736(95)92474-4

[22] Devlieger R, D’Hooghe T, Timmerman D (2003) Uterine adenomyosis in the infertility clinic. Hum Reprod Update 9:139–47 12751776 10.1093/humupd/dmg010

[23] Uduwela AS, Perera MA, Aiqing L, Fraser IS (2000) Endometrial-myometrial interface: relationship to adenomyosis and changes in pregnancy. Obstet Gynecol Surv 55:390–400 10841317 10.1097/00006254-200006000-00025

[24] Zhai J, Vannuccini S, Petraglia F, Giudice LC (2020) Adenomyosis: Mechanisms and Pathogenesis. Semin Reprod Med 38:129–143 33032339 10.1055/s-0040-1716687 PMC7932680

[25] Leyendecker G, Bilgicyildirim A, Inacker M, Stalf T, Huppert P, Mall G, Böttcher B, Wildt L (2015) Adenomyosis and endometriosis. Re-visiting their association and further insights into the mechanisms of auto-traumatisation. An MRI study. Arch Gynecol Obstet 291:917–32 25241270 10.1007/s00404-014-3437-8 PMC4355446

[26] Vannuccini S, Tosti C, Carmona F, Huang SJ, Chapron C, Guo S-W, Petraglia F (2017) Pathogenesis of adenomyosis: an update on molecular mechanisms. Reprod Biomed Online 35:592–601 28693952 10.1016/j.rbmo.2017.06.016

[27] Yang G, Im H-J, Wang JHC (2005) Repetitive mechanical stretching modulates IL-1β induced COX-2, MMP-1 expression, and PGE2 production in human patellar tendon fibroblasts. Gene 363:166–172 16226404 10.1016/j.gene.2005.08.006 PMC2901527

[28] Noble LS, Takayama K, Zeitoun KM, Putman JM, Johns DA, Hinshelwood MM, Agarwal VR, Zhao Y, Carr BR, Bulun SE (1997) Prostaglandin E2 Stimulates Aromatase Expression in Endometriosis-Derived Stromal Cells*. J Clin Endocrinol Metab 82:600–6 9024261 10.1210/jcem.82.2.3783

[29] Chen Y-J, Li H-Y, Chang Y-L, Yuan C-C, Tai L-K, Lu KH, Chang C-M, Chiou S-H (2010) Suppression of migratory/invasive ability and induction of apoptosis in adenomyosis-derived mesenchymal stem cells by cyclooxygenase-2 inhibitors. Fertil Steril 94:1972–9.e4 20227073 10.1016/j.fertnstert.2010.01.070

[30] Carrarelli P, Yen C-F, Funghi L, Arcuri F, Tosti C, Bifulco G, Luddi A, Lee C-L, Petraglia F (2017) Expression of Inflammatory and Neurogenic Mediators in Adenomyosis: A Pathogenetic Role. Reprod Sci 24:369–375 27440813 10.1177/1933719116657192

[31] Donnez O, Donnez J (2020) Gonadotropin-releasing hormone antagonist (linzagolix): a new therapy for uterine adenomyosis. Fertil Steril 114:640–645 32507315 10.1016/j.fertnstert.2020.04.017

[32] Donnez J, Donnez O, Tourniaire J, Brethous M, Bestel E, Garner E, Charpentier S, Humberstone A, Loumaye E (2021) Uterine adenomyosis treated by Linzagolix, an oral gonadotropin-releasing hormone receptor antagonist: a pilot study with a new “Hit Hard First and then Maintain” regimen of administration. J Clin Med. 10.3390/jcm10245794 34945090 10.3390/jcm10245794 PMC8706704

[33] Bofill Rodriguez M, Lethaby A, Farquhar C (2019) Non-steroidal anti-inflammatory drugs for heavy menstrual bleeding. Cochrane Database Syst Rev 9:Cd000400 31535715 10.1002/14651858.CD000400.pub4 PMC6751587

[34] Marjoribanks J, Ayeleke RO, Farquhar C, Proctor M (2015) Nonsteroidal anti-inflammatory drugs for dysmenorrhoea. *Cochrane Database Syst Rev* 2015: Cd001751 10.1002/14651858.CD001751.pub3 PMC6953236 26224322

[35] Taylor AH, Kalathy V, Habiba M (2014) Estradiol and tamoxifen enhance invasion of endometrial stromal cells in a three-dimensional coculture model of adenomyosis. Fertil Steril 101:288–293 24188882 10.1016/j.fertnstert.2013.09.042

[36] Zeng YY, Guan YG, Li KY (2017) [Role of estrogen, estrogen receptors, and aromatase in the pathogenesis of uterine adenomyosis]. Nan Fang Yi Ke Da Xue Xue Bao 37:383–7 28377357 10.3969/j.issn.1673-4254.2017.03.18 PMC6780442

[37] Sztachelska M, Ponikwicka-Tyszko D, Martínez-Rodrigo L, Bernaczyk P, Palak E, Półchłopek W, Bielawski T, Wołczyński S (2022) Functional implications of estrogen and progesterone receptors expression in adenomyosis, potential targets for endocrinological therapy. J Clin Med. 10.3390/jcm11154407 35956024 10.3390/jcm11154407 PMC9369051

[38] Huang JH, Duan H, Wang S, Wang YY (2021) Estrogen 17β‑estradiol accelerates the proliferation of uterine junctional zone smooth muscle cells via the let‑7a/Lin28B axis in adenomyosis. Mol Med Rep. 10.3892/mmr.2021.11976 34227673 10.3892/mmr.2021.11976

[39] Chen YJ, Li HY, Huang CH, Twu NF, Yen MS, Wang PH, Chou TY, Liu YN, Chao KC, Yang MH (2010) Oestrogen-induced epithelial-mesenchymal transition of endometrial epithelial cells contributes to the development of adenomyosis. J Pathol 222:261–270 20814901 10.1002/path.2761

[40] Mai KT, Yazdi HM, Perkins DG, Parks W (1997) Pathogenetic role of the stromal cells in endometriosis and adenomyosis. Histopathology 30:430–442 9181364 10.1046/j.1365-2559.1997.4910725.x

[41] Yildiz S, Kinali M, Wei JJ, Milad M, Yin P, Adli M, Bulun SE (2023) Adenomyosis: single-cell transcriptomic analysis reveals a paracrine mesenchymal-epithelial interaction involving the WNT/SFRP pathway. Fertil Steril 119:869–882 36736810 10.1016/j.fertnstert.2023.01.041 PMC11257082

[42] He X, Liu N, Mu T, Lu D, Jia C, Wang S, Yin C, Liu L, Zhou L, Huang X, Ma Y (2020) Oestrogen induces epithelial-mesenchymal transition in endometriosis via circ_0004712/miR-148a-3p sponge function. J Cell Mol Med 24:9658–9666 32667746 10.1111/jcmm.15495 PMC7520264

[43] Huang TS, Chen YJ, Chou TY, Chen CY, Li HY, Huang BS, Tsai HW, Lan HY, Chang CH, Twu NF, Yen MS, Wang PH, Chao KC, Lee CC, Yang MH (2014) Oestrogen-induced angiogenesis promotes adenomyosis by activating the Slug-VEGF axis in endometrial epithelial cells. J Cell Mol Med 18:1358–1371 24758741 10.1111/jcmm.12300 PMC4124020

[44] Zhang Z, Qin Y, Huang J, Wang Y, Zeng L, Wang Y, Zhuyun F, Wang L (2024) Oestrogen promotes the progression of adenomyosis by inhibiting < em>CITED2 through miR-145. Reprod Biomed Online 49 10.1016/j.rbmo.2024.104108 39293195

[45] Yamamoto T, Noguchi T, Tamura T, Kitawaki J, Okada H (1993) Evidence for estrogen synthesis in adenomyotic tissues. Am J Obstet Gynecol 169:734–738 8372890 10.1016/0002-9378(93)90654-2

[46] Takahashi K, Nagata H, Kitao M (1989) Clinical usefulness of determination of estradiol level in the menstrual blood for patients with endometriosis. Nihon Sanka Fujinka Gakkai Zasshi 41:1849–1850 2592808

[47] Wang Y, Qu Y, Song W (2015) Genetic variation in COX-2 -1195 and the risk of endometriosis and adenomyosis. 42:168–172 26054111

[48] Tong X, Li Z, Wu Y, Fu X, Zhang Y, Fan H (2014) COMT 158G/A and CYP1B1 432 C/G polymorphisms increase the risk of endometriosis and adenomyosis: a meta-analysis. Eur J Obstet Gynecol Reprod Biol 179:17–21 24965973 10.1016/j.ejogrb.2014.04.039

[49] Kitawaki J, Kado N, Ishihara H, Koshiba H, Kitaoka Y, Honjo H (2002) Endometriosis: the pathophysiology as an estrogen-dependent disease. J Steroid Biochem Mol Biol 83:149–155 12650711 10.1016/s0960-0760(02)00260-1

[50] Lai ZZ, Yang HL, Ha SY, Chang KK, Mei J, Zhou WJ, Qiu XM, Wang XQ, Zhu R, Li DJ, Li MQ (2019) Cyclooxygenase-2 in endometriosis. Int J Biol Sci 15:2783–2797 31853218 10.7150/ijbs.35128 PMC6909960

[51] Ji F, Yang X, He Y, Wang H, Aili A, Ding Y (2017) Aberrant endometrial DNA methylome of homeobox A10 and catechol-O-methyltransferase in endometriosis. J Assist Reprod Genet 34:409–415 28074437 10.1007/s10815-016-0862-6 PMC5360687

[52] Johnson MC, Pinto C, Alves A, Palomino A, Fuentes A, Boric MA, Vega M (2004) [P450Arom and estrogenic microenvironment of eutopic endometria in endometriosis]. Rev Med Chil 132:1475–82 15743158 10.4067/s0034-98872004001200004

[53] Kitawaki J, Noguchi T, Amatsu T, Maeda K, Tsukamoto K, Yamamoto T, Fushiki S, Osawa Y, Honjo H (1997) Expression of aromatase cytochrome P450 protein and messenger ribonucleic acid in human endometriotic and adenomyotic tissues but not in normal endometrium. Biol Reprod 57:514–519 9282984 10.1095/biolreprod57.3.514

[54] Fang Z, Yang S, Gurates B, Tamura M, Simpson E, Evans D, Bulun SE (2002) Genetic or enzymatic disruption of aromatase inhibits the growth of ectopic uterine tissue. J Clin Endocrinol Metab 87:3460–3466 12107266 10.1210/jcem.87.7.8683

[55] Kitawaki J, Kusuki I, Koshiba H, Tsukamoto K, Fushiki S, Honjo H (1999) Detection of aromatase cytochrome P-450 in endometrial biopsy specimens as a diagnostic test for endometriosis. Fertil Steril 72:1100–1106 10593388 10.1016/s0015-0282(99)00424-0

[56] Visnovský J, Galo S, Zúbor P, Hatok J, Racay P, Danko J (2008) [Semiquantitative analysis of mRNA aromatase expression in eutopic endometrium as a diagnostic marker of endometriosis and estrogen dependent diseases]. Ceska Gynekol 73:213–7 18711959

[57] Li C, Chen R, Jiang C, Chen L, Cheng Z (2019) Correlation of LOX–5 and COX–2 expression with inflammatory pathology and clinical features of adenomyosis. Mol Med Rep 19:727–733 30387822 10.3892/mmr.2018.9618

[58] Maia H Jr., Haddad C, Coelho G, Casoy J (2012) Role of inflammation and aromatase expression in the eutopic endometrium and its relationship with the development of endometriosis. Womens Health (Lond) 8:647–58 23181530 10.2217/whe.12.52

[59] Attar E, Bulun SE (2006) Aromatase and other steroidogenic genes in endometriosis: translational aspects. Hum Reprod Update 12:49–56 16123052 10.1093/humupd/dmi034

[60] Sieiński W (1993) Tumor-like intravascular proliferations of the stroma in adenomyosis. Patol Pol 44:1–4 8488076

[61] Thiery JP (2009) [Epithelial-mesenchymal transitions in cancer onset and progression]. Bull Acad Natl Med 193:1969–78 20666011

[62] Goteri G, Lucarini G, Montik N, Zizzi A, Stramazzotti D, Fabris G, Tranquilli AL, Ciavattini A (2009) Expression of vascular endothelial growth factor (VEGF), hypoxia inducible factor-1alpha (HIF-1alpha), and microvessel density in endometrial tissue in women with adenomyosis. Int J Gynecol Pathol 28:157–163 19188818 10.1097/PGP.0b013e318182c2be

[63] Zhou S, Yi T, Liu R, Bian C, Qi X, He X, Wang K, Li J, Zhao X, Huang C, Wei Y (2012) Proteomics identification of annexin A2 as a key mediator in the metastasis and proangiogenesis of endometrial cells in human adenomyosis. Mol Cell Proteomics 11:M112.017988 22493182 10.1074/mcp.M112.017988 PMC3394960

[64] Wang YY, Duan H, Wang S, Quan YJ, Huang JH, Guo ZC (2021) Upregulated Talin1 synergistically boosts β-estradiol-induced proliferation and pro-angiogenesis of eutopic and ectopic endometrial stromal cells in adenomyosis. Reprod Biol Endocrinol 19:70 33990206 10.1186/s12958-021-00756-7 PMC8120781

[65] Wang YY, Duan H, Wang S, Quan YJ, Huang JH, Guo ZC (2021) Talin1 induces epithelial-mesenchymal transition to facilitate endometrial cell migration and invasion in Adenomyosis under the regulation of microRNA-145-5p. Reprod Sci 28:1523–1539 33537874 10.1007/s43032-020-00444-8

[66] Zheng D, Duan H, Wang S, Xu Q, Gan L, Li J, Dong Q (2018) FAK regulates epithelial–mesenchymal transition in Adenomyosis. Mol Med Rep 18:5461–5472 30365102 10.3892/mmr.2018.9600 PMC6236295

[67] Haydari Z, Shams H, Jahed Z, Mofrad MRK (2020) Kindlin assists Talin to promote Integrin activation. Biophys J 118:1977–1991 32191864 10.1016/j.bpj.2020.02.023 PMC7175420

[68] Pulous FE, Grimsley-Myers CM, Kansal S, Kowalczyk AP, Petrich BG (2019) Talin-dependent Integrin activation regulates VE-Cadherin localization and endothelial cell barrier function. Circ Res 124:891–903 30707047 10.1161/CIRCRESAHA.118.314560 PMC6521868

[69] Di Cristofano A (2017) SGK1: the dark side of PI3K signaling. Curr Top Dev Biol 123:49–71 28236975 10.1016/bs.ctdb.2016.11.006 PMC5658788

[70] Zhang J, Zhou Y, Huang T, Wu F, Pan Y, Dong Y, Wang Y, Chan AKY, Liu L, Kwan JSH, Cheung AHK, Wong CC, Lo AKF, Cheng ASL, Yu J, Lo KW, Kang W, To KF (2019) FGF18, a prominent player in FGF signaling, promotes gastric tumorigenesis through autocrine manner and is negatively regulated by miR-590-5p. Oncogene 38:33–46 30082912 10.1038/s41388-018-0430-x PMC6318220

[71] Chen T, Gong W, Tian H, Wang H, Chu S, Ma J, Yang H, Cheng J, Liu M, Li X, Jiang C (2017) Fibroblast growth factor 18 promotes proliferation and migration of H460 cells via the ERK and p38 signaling pathways. Oncol Rep 37:1235–1242 27959447 10.3892/or.2016.5301

[72] Wu J, Tao X, Zhang H, Yi XH, Yu YH (2020) Estrogen-induced stromal FGF18 promotes proliferation and invasion of endometrial carcinoma cells through ERK and Akt signaling. Cancer Manag Res 12:6767–6777 32801905 10.2147/CMAR.S254242 PMC7414926

[73] Orlacchio A, Ranieri M, Brave M, Arciuch VA, Forde T, De Martino D, Anderson KE, Hawkins P, Di Cristofano A (2017) SGK1 is a critical component of an AKT-independent pathway essential for PI3K-mediated tumor development and maintenance. Cancer Res 77:6914–26 29055016 10.1158/0008-5472.CAN-17-2105 PMC5732884

[74] Sommer EM, Dry H, Cross D, Guichard S, Davies BR, Alessi DR (2013) Elevated SGK1 predicts resistance of breast cancer cells to Akt inhibitors. Biochem J 452:499–508 23581296 10.1042/BJ20130342 PMC3671793

[75] Wu Y, Wang H, Li Y, Li Y, Liang Y, Zhong G, Zhang Q (2022) Estrogen-increased SGK1 promotes endometrial stromal cell invasion in adenomyosis by regulating with LPAR2. Reprod Sci 29:3026–3038 35799024 10.1007/s43032-022-00990-3

[76] Lou Y, Fu Z, Tian Y, Hu M, Wang Q, Zhou Y, Wang N, Zhang Q, Jin F (2023) Estrogen-sensitive activation of SGK1 induces M2 macrophages with anti-inflammatory properties and a Th2 response at the maternal-fetal interface. Reprod Biol Endocrinol 21:50 37226177 10.1186/s12958-023-01102-9 PMC10207684

[77] Zhu H, Jiang X, Zhou X, Dong X, Xie K, Yang C, Jiang H, Sun X, Lu J (2018) Neuropilin-1 regulated by miR-320 contributes to the growth and metastasis of cholangiocarcinoma cells. Liver Int 38:125–135 28618167 10.1111/liv.13495

[78] Kiso M, Tanaka S, Saji S, Toi M, Sato F (2018) Long isoform of VEGF stimulates cell migration of breast cancer by filopodia formation via NRP1/ARHGAP17/Cdc42 regulatory network. Int J Cancer 143:2905–2918 29971782 10.1002/ijc.31645 PMC6282968

[79] Hu R, Peng GQ, Ban DY, Zhang C, Zhang XQ, Li YP (2020) High-expression of Neuropilin 1 correlates to estrogen-induced epithelial-mesenchymal transition of endometrial cells in adenomyosis. Reprod Sci 27:395–403 32046395 10.1007/s43032-019-00035-2

[80] Huang N, Xu L, Qiu Y, Zhan J, Chen X (2021) Down-regulated miR-124-3p enhanced the migration and epithelial-stromal transformation of endometrial stromal cells extracted from eutopic endometrium in subjects with adenomyosis by up-regulating Neuropilin 1. Tissue Cell 69:101474 33387827 10.1016/j.tice.2020.101474

[81] Li J, Yanyan M, Mu L, Chen X, Zheng W (2019) The expression of Bcl-2 in adenomyosis and its effect on proliferation, migration, and apoptosis of endometrial stromal cells. Pathology 215:152477 10.1016/j.prp.2019.152477 31174926

[82] Orazov M, Radzinsky V, Sharapova O, Kostin I, Chitanava Y (2020) Oxytocinergic regulation in pathogenesis of pelvic pain caused by adenomyosis. Gynecol Endocrinol 36:20–23 33305666 10.1080/09513590.2020.1816723

[83] Fleming JG, Spencer TE, Safe SH, Bazer FW (2006) Estrogen regulates transcription of the ovine oxytocin receptor gene through GC-rich SP1 promoter elements. Endocrinology 147:899–911 16254027 10.1210/en.2005-1120

[84] Kitawaki J (2006) Adenomyosis: the pathophysiology of an oestrogen-dependent disease. Best Pract Res Clin Obstet Gynaecol 20:493–502 16564227 10.1016/j.bpobgyn.2006.01.010

[85] Wilson T, Liggins GC, Whittaker DJ (1988) Oxytocin stimulates the release of arachidonic acid and prostaglandin F_2_ alpha from human decidual cells. Prostaglandins 35:771–780 2840690 10.1016/0090-6980(88)90149-9

[86] Burns PD, Mendes JO Jr., Yemm RS, Clay CM, Nelson SE, Hayes SH, Silvia WJ (2001) Cellular mechanisms by which oxytocin mediates ovine endometrial prostaglandin F_2_alpha synthesis: role of G(i) proteins and mitogen-activated protein kinases. Biol Reprod 65:1150–5 11566737 10.1095/biolreprod65.4.1150

[87] Sales KJ, Jabbour HN (2003) Cyclooxygenase enzymes and prostaglandins in pathology of the endometrium. Reproduction 126:559–67 14611628 10.1530/rep.0.1260559 PMC2695735

[88] Sales KJ, Grant V, Jabbour HN (2008) Prostaglandin E2 and F2alpha activate the FP receptor and up-regulate cyclooxygenase-2 expression via the cyclic AMP response element. Mol Cell Endocrinol 285:51–61 18316157 10.1016/j.mce.2008.01.016 PMC2694994

[89] Shi J, Jin L, Leng J, Lang J (2015) [Response of potassium channels to estrogen and progesterone in the uterine smooth muscle cells of adenomyosis in vitro]. Zhonghua Fu Chan Ke Za Zhi 50:843–7 26887773

[90] Wang S, Duan H, Zhang Y, Wang L, Zhang H, Li G (2015) [Mechanism of 17β-estrogen on intracellular free calcium regulation in smooth muscle cells at the endometrial-myometrial interface in uteri with adenomyosis]. Zhonghua Fu Chan Ke Za Zhi 50:510–5 26311641

[91] Wang S, Duan H (2015) Rapid effects of estrogen on intracellular Ca(2+) regulation in junctional myometrium through the menstrual cycle in uteri with and without adenomyosis. J Minim Invasive Gynecol 22:S173 27678945 10.1016/j.jmig.2015.08.642

[92] Wang S, Duan H, Li B (2020) Rapid effects of oestrogen on intracellular Ca(2+) in the uterine junctional myometrium of patients with and without adenomyosis in different phases of the menstrual cycle. Reprod Sci 27:1992–2001 32542538 10.1007/s43032-020-00218-2

[93] Ghasemi A, Hashemy SI, Aghaei M, Panjehpour M (2017) RhoA/ROCK pathway mediates leptin-induced uPA expression to promote cell invasion in ovarian cancer cells. Cell Signal 32:104–114 28104444 10.1016/j.cellsig.2017.01.020

[94] Jiang QY, Xia JM, Ding HG, Fei XW, Lin J, Wu RJ (2012) RNAi-mediated blocking of ezrin reduces migration of ectopic endometrial cells in endometriosis. Mol Hum Reprod 18:435–441 22544491 10.1093/molehr/gas019

[95] Wang S, Duan H, Zhang Y, Liu JJ (2013) [Expression of RhoA and Rho kinase in junctional zone of human adenomyosis and its relationship with dysmenorrheal]. Zhonghua Fu Chan Ke Za Zhi 48:911–5 24495684

[96] Wang S, Duan H, Zhang Y, Sun FQ (2016) Abnormal activation of RhoA/ROCK-I signaling in junctional zone smooth muscle cells of patients with adenomyosis. Reprod Sci 23:333–341 26335177 10.1177/1933719115602764

[97] Sun FQ, Duan H, Wang S, Li JJ (2015) 17β-Estradiol induces overproliferation in adenomyotic human uterine smooth muscle cells of the junctional zone through hyperactivation of the Estrogen Receptor-enhanced RhoA/ROCK signaling pathway. Reprod Sci 22:1436–1444 25940707 10.1177/1933719115584447

[98] Kobayashi H (2023) Molecular targets for nonhormonal treatment based on a multistep process of adenomyosis development. Reprod Sci 30:743–60 35838920 10.1007/s43032-022-01036-4

[99] Benagiano G, Brosens I, Habiba M (2014) Structural and molecular features of the endomyometrium in endometriosis and adenomyosis. Hum Reprod Update 20:386–402 24140719 10.1093/humupd/dmt052

[100] Khan KN, Fujishita A, Suematsu T, Ogawa K, Koshiba A, Mori T, Itoh K, Teramukai S, Matsuda K, Nakashima M, Kitawaki J (2021) An axonemal alteration in apical endometria of human adenomyosis. Hum Reprod 36:1574–1589 33889963 10.1093/humrep/deab090

[101] Shi J, Xu Q, Yu S, Zhang T (2025) Perturbations of the endometrial immune microenvironment in endometriosis and adenomyosis: their impact on reproduction and pregnancy. Semin Immunopathol 47:16 39966111 10.1007/s00281-025-01040-1 PMC11835911

[102] Maybin JA, Critchley HO (2015) Menstrual physiology: implications for endometrial pathology and beyond. Hum Reprod Update 21:748–761 26253932 10.1093/humupd/dmv038 PMC4594618

[103] Kaunitz AM (2000) Menstruation: choosing whether...and when. Contraception 62:277–84 11239613 10.1016/s0010-7824(00)00182-7

[104] Vannuccini S, Clifton VL, Fraser IS, Taylor HS, Critchley H, Giudice LC, Petraglia F (2016) Infertility and reproductive disorders: impact of hormonal and inflammatory mechanisms on pregnancy outcome. Hum Reprod Update 22:104–115 26395640 10.1093/humupd/dmv044 PMC7289323

[105] McKinnon BD, Bertschi D, Bersinger NA, Mueller MD (2015) Inflammation and nerve fiber interaction in endometriotic pain. Trends Endocrinol Metab 26:1–10 25465987 10.1016/j.tem.2014.10.003

[106] Halis G, Arici A (2004) Endometriosis and inflammation in infertility. Ann N Y Acad Sci 1034:300–315 15731321 10.1196/annals.1335.032

[107] Lin YH, Chen YH, Chang HY, Au HK, Tzeng CR, Huang YH (2018) Chronic niche inflammation in endometriosis-associated infertility: current understanding and future therapeutic strategies. Int J Mol Sci. 10.3390/ijms19082385 30104541 10.3390/ijms19082385 PMC6121292

[108] Huang Q, Yu Y, Xu W, Li S, Zhou Y, Shu J (2023) The role of peritoneal immunity in peritoneal endometriosis and related infertility. Front Biosci (Landmark Ed) 28:166 37664916 10.31083/j.fbl2808166

[109] Mikuš M, Goldštajn M, Brlečić I, Dumančić S, Laganà AS, Chiantera V, Vujić G, Ćorić M (2022) CTLA4-Linked autoimmunity in the pathogenesis of endometriosis and related infertility: a systematic review. Int J Mol Sci. 10.3390/ijms231810902 36142815 10.3390/ijms231810902 PMC9504308

[110] Kolanska K, Alijotas-Reig J, Cohen J, Cheloufi M, Selleret L, d’Argent E, Kayem G, Valverde EE, Fain O, Bornes M, Darai E, Mekinian A (2021) Endometriosis with infertility: a comprehensive review on the role of immune deregulation and immunomodulation therapy. Am J Reprod Immunol 85:e13384 33278837 10.1111/aji.13384

[111] Wang XM, Ma ZY, Song N (2018) Inflammatory cytokines IL-6, IL-10, IL-13, TNF-α and peritoneal fluid flora were associated with infertility in patients with endometriosis. Eur Rev Med Pharmacol Sci 22:2513–2518 29771400 10.26355/eurrev_201805_14899

[112] Khan KN, Masuzaki H, Fujishita A, Kitajima M, Hiraki K, Sekine I, Matsuyama T, Ishimaru T (2005) Interleukin-6- and tumour necrosis factor alpha-mediated expression of hepatocyte growth factor by stromal cells and its involvement in the growth of endometriosis. Hum Reprod 20:2715–2723 16006475 10.1093/humrep/dei156

[113] Propst AM, Quade BJ, Nowak RA, Stewart EA (2002) Granulocyte macrophage colony-stimulating factor in adenomyosis and autologous endometrium. J Soc Gynecol Investig 9:93–97 11963838 10.1016/s1071-5576(01)00160-5

[114] Stratopoulou CA, Camboni A, Donnez J, Dolmans MM (2021) Identifying common pathogenic features in deep endometriotic nodules and uterine adenomyosis. J Clin Med. 10.3390/jcm10194585 34640603 10.3390/jcm10194585 PMC8509556

[115] Wang B, Yang Y, Deng X, Ban Y, Chao L (2020) Interaction of M2 macrophages and endometrial cells induces downregulation of GRIM-19 in endometria of adenomyosis. Reprod Biomed Online 41:790–800 32896475 10.1016/j.rbmo.2020.04.022

[116] Stratopoulou CA, Cussac S, d’Argent M, Donnez J, Dolmans MM (2023) M2 macrophages enhance endometrial cell invasiveness by promoting collective cell migration in uterine adenomyosis. Reprod Biomed Online 46:729–38 36792417 10.1016/j.rbmo.2023.01.001

[117] An M, Li D, Yuan M, Li Q, Zhang L, Wang G (2017) Different macrophages equally induce EMT in endometria of adenomyosis and normal. Reproduction 154:79–92 28495851 10.1530/REP-17-0174

[118] An M, Li D, Yuan M, Li Q, Zhang L, Wang G (2017) Interaction of macrophages and endometrial cells induces epithelial-mesenchymal transition-like processes in adenomyosis. Biol Reprod 96:46–57 28395325 10.1095/biolreprod.116.144071

[119] Hu Y, Yuan M, Cheng L, Wang G (2023) Extracellular vesicles contribute to EMT in adenomyosis by inducing macrophage polarization†. Biol Reprod 108:584–96 36721984 10.1093/biolre/ioad015

[120] Bourdon M, Santulli P, Doridot L, Jeljeli M, Chêne C, Chouzenoux S, Nicco C, Marcellin L, Chapron C, Batteux F (2021) Immune cells and Notch1 signaling appear to drive the epithelial to mesenchymal transition in the development of adenomyosis in mice. Mol Hum Reprod. 10.1093/molehr/gaab053 34463756 10.1093/molehr/gaab053

[121] Ribatti D, Tamma R, Annese T (2020) Mast cells and angiogenesis in multiple sclerosis. Inflamm Res 69:1103–1110 32808153 10.1007/s00011-020-01394-2

[122] Komi DEA, Redegeld FA (2020) Role of mast cells in shaping the tumor microenvironment. Clin Rev Allergy Immunol 58:313–325 31256327 10.1007/s12016-019-08753-w PMC7244463

[123] Che X, Wang J, He J, Guo X, Li T, Zhang X (2020) The new application of mifepristone in the relief of adenomyosis-caused dysmenorrhea. Int J Med Sci 17:224–233 32038106 10.7150/ijms.39252 PMC6990887

[124] Istrate-Ofiţeru AM, Pîrvan IC, Pirici D, Roşu GC, Niculescu M, Berceanu S, Manolea MM, Comănescu MV, Voicu NL, Iovan L, Vasile MM, Căpitănescu RG, Diţescu D, Mogoantă L, Berceanu C (2019) Triple immunohistochemistry for assessing the inflammatory, vascular and progression of adenomyosis. Rom J Morphol Embryol 60:419–428 31658314

[125] Niu W, Zhang Y, Liu H, Liang N, Xu L, Li Y, Yao W, Shi W, Liu Z (2023) Single-cell profiling uncovers the roles of endometrial fibrosis and microenvironmental changes in adenomyosis. J Inflamm Res 16:1949–1965 37179754 10.2147/JIR.S402734 PMC10167994

[126] Pan MS, Wang H, Ansari KH, Li XP, Sun W, Fan YZ (2020) Gallbladder cancer-associated fibroblasts promote vasculogenic mimicry formation and tumor growth in gallbladder cancer via upregulating the expression of NOX4, a poor prognosis factor, through IL-6-JAK-STAT3 signal pathway. J Exp Clin Cancer Res 39:234 33153467 10.1186/s13046-020-01742-4 PMC7643415

[127] Hu X, Li J, Fu M, Zhao X, Wang W (2021) The JAK/STAT signaling pathway: from bench to clinic. Signal Transduct Target Ther 6:402 34824210 10.1038/s41392-021-00791-1 PMC8617206

[128] Herington JL, Bruner-Tran KL, Lucas JA, Osteen KG (2011) Immune interactions in endometriosis. Expert Rev Clin Immunol 7:611–626 21895474 10.1586/eci.11.53 PMC3204940

[129] Maurer M, Metz M (2005) The status quo and quo vadis of mast cells. Exp Dermatol 14:923–929 16274460 10.1111/j.1600-0625.2005.00369.x

[130] Galli SJ, Grimbaldeston M, Tsai M (2008) Immunomodulatory mast cells: negative, as well as positive, regulators of immunity. Nat Rev Immunol 8:478–486 18483499 10.1038/nri2327 PMC2855166

[131] Dudeck A, Suender CA, Kostka SL, von Stebut E, Maurer M (2011) Mast cells promote Th1 and Th17 responses by modulating dendritic cell maturation and function. Eur J Immunol 41:1883–1893 21491417 10.1002/eji.201040994

[132] Osuga Y, Koga K, Hirota Y, Hirata T, Yoshino O, Taketani Y (2011) Lymphocytes in endometriosis. Am J Reprod Immunol 65:1–10 20584009 10.1111/j.1600-0897.2010.00887.x

[133] Saito A, Osuga Y, Yoshino O, Takamura M, Hirata T, Hirota Y, Koga K, Harada M, Takemura Y, Yano T, Taketani Y (2011) TGF-β1 induces proteinase-activated receptor 2 (PAR2) expression in endometriotic stromal cells and stimulates PAR2 activation-induced secretion of IL-6. Hum Reprod 26:1892–1898 21546388 10.1093/humrep/der125

[134] Bulmer JN, Jones RK, Searle RF (1998) Intraepithelial leukocytes in endometriosis and adenomyosis: comparison of eutopic and ectopic endometrium with normal endometrium. Hum Reprod 13:2910–2915 9804254 10.1093/humrep/13.10.2910

[135] Liu W, Sheng S, Zhu C, Li C, Zou Y, Yang C, Chen ZJ, Wang F, Jiao X (2023) Increased NKG2A(+)CD8(+) T-cell exhaustion in patients with adenomyosis. Mucosal Immunol 16:121–134 36828189 10.1016/j.mucimm.2023.02.003

[136] Tang R, Rangachari M, Kuchroo VK (2019) Tim-3: a co-receptor with diverse roles in T cell exhaustion and tolerance. Semin Immunol 42:101302 31604535 10.1016/j.smim.2019.101302

[137] Gorman JV, Colgan JD (2014) Regulation of T cell responses by the receptor molecule Tim-3. Immunol Res 59:56–65 24825777 10.1007/s12026-014-8524-1 PMC4125508

[138] Huang P, Lv C, Zhang C, Feng H, Li C, Zhang H, Zhao X, Li M (2020) Expression and significance of T-cell immunoglobulin mucin molecule 3 and its ligand galectin-9 in patients with adenomyosis. Gynecol Endocrinol 36:605–610 32319832 10.1080/09513590.2020.1754788

[139] Gui T, Chen C, Zhang Z, Tang W, Qian R, Ma X, Cao P, Wan G (2014) The disturbance of T_H_17-Treg cell balance in adenomyosis. Fertil Steril 101:506–14 24331831 10.1016/j.fertnstert.2013.10.050

[140] Yang J-H, Chen M-J, Wu M-Y, Chen Y-C, Yang Y-S, Ho H-N (2006) Decreased suppression of interleukin-6 after treatment with medroxyprogesterone acetate and danazol in endometrial stromal cells of women with adenomyosis. Fertil Steril 86:1459–1465 16989825 10.1016/j.fertnstert.2006.04.034

[141] Yang JH, Wu MY, Chang DY, Chang CH, Yang YS, Ho HN (2006) Increased interleukin-6 messenger RNA expression in macrophage-cocultured endometrial stromal cells in adenomyosis. Am J Reprod Immunol 55:181–187 16451352 10.1111/j.1600-0897.2005.00363.x

[142] Bettelli E, Carrier Y, Gao W, Korn T, Strom TB, Oukka M, Weiner HL, Kuchroo VK (2006) Reciprocal developmental pathways for the generation of pathogenic effector TH17 and regulatory t cells. Nature 441:235–238 16648838 10.1038/nature04753

[143] Ivanov II, McKenzie BS, Zhou L, Tadokoro CE, Lepelley A, Lafaille JJ, Cua DJ, Littman DR (2006) The orphan nuclear receptor RORgammat directs the differentiation program of proinflammatory IL-17 + t helper cells. Cell 126:1121–1133 16990136 10.1016/j.cell.2006.07.035

[144] Sikora J, Smycz-Kubańska M, Mielczarek-Palacz A, Bednarek I, Kondera-Anasz Z (2018) The involvement of multifunctional TGF-β and related cytokines in pathogenesis of endometriosis. Immunol Lett 201:31–37 30367890 10.1016/j.imlet.2018.10.011

[145] Miller JE, Ahn SH, Marks RM, Monsanto SP, Fazleabas AT, Koti M, Tayade C (2020) IL-17A modulates peritoneal macrophage recruitment and M2 polarization in endometriosis. Front Immunol 11:108 32117261 10.3389/fimmu.2020.00108 PMC7034338

[146] Takamura M, Osuga Y, Izumi G, Yoshino O, Koga K, Saito A, Hirata T, Hirota Y, Harada M, Hasegawa A, Taketani Y (2012) Interleukin-17A is present in neutrophils in endometrioma and stimulates the secretion of growth-regulated oncogene-α (Gro-α) from endometrioma stromal cells. Fertil Steril 98:1218–24e1 22902060 10.1016/j.fertnstert.2012.07.1117

[147] Hsu L-T, Lu P-C, Wang Y-W, Wu H-M, Chen I-J, Huang H-Y (2024) Eutopic and ectopic endometrial interleukin-17 and interleukin-17 receptor expression at the endometrial—myometrial interface in women with adenomyosis: Possible pathophysiology implications. Int J Mol Sci 25:11155 39456936 10.3390/ijms252011155 PMC11508639

[148] Yang J-H, Chen M-J, Chen H-F, Lee T-H, Ho H-N, Yang Y-S (2004) Decreased expression of killer cell inhibitory receptors on natural killer cells in eutopic endometrium in women with adenomyosis. Hum Reprod 19:1974–1978 15217996 10.1093/humrep/deh372

[149] Chen Y, Zhu J, Chen L, Shen Y, Zhang J, Wang Q (2022) SFRP4(+)IGFBP5(hi) NKT cells induced neural-like cell differentiation to contribute to adenomyosis pain. Front Immunol 13:945504 36532077 10.3389/fimmu.2022.945504 PMC9750790

[150] Umesaki N, Tanaka T, Miyama M, Mizuno K, Kawamura N, Ogita S (1999) Increased natural killer cell activities in patients treated with gonadotropin releasing hormone agonist. Gynecol Obstet Invest 48:66–68 10394096 10.1159/000010137

[151] Prathoomthong S, Tingthanatikul Y, Lertvikool S, Rodratn N, Waiyaput W, Dittharot K, Sroyraya M, Sophonsritsuk A (2018) The effects of Dienogest on macrophage and natural killer cells in adenomyosis: a randomized controlled study. Int J Fertil Steril 11:279–286 29043703 10.22074/ijfs.2018.5137 PMC5641459

[152] Orazov MR, Radzinskiy VE, Nosenko OM (2016) [The role of inflammatory and immune reactivity in developing pain in adenomyosis ]. Patol Fiziol Eksp Ter 60:40–4 29215246

[153] Wei Y, Liang Y, Lin H, Dai Y, Yao S (2020) Autonomic nervous system and inflammation interaction in endometriosis-associated pain. J Neuroinflammation 17:80 32145751 10.1186/s12974-020-01752-1 PMC7060607

[154] Sreya M, Tucker DR, Yi J, Alotaibi FT, Lee AF, Noga H, Yong PJ (2024) Nerve bundle density and expression of NGF and IL-1β are intra-individually heterogenous in subtypes of endometriosis. Biomolecules. 10.3390/biom14050583 38785989 10.3390/biom14050583 PMC11118880

[155] Bourdon M, Santulli P, Jeljeli M, Vannuccini S, Marcellin L, Doridot L, Petraglia F, Batteux F, Chapron C (2021) Immunological changes associated with adenomyosis: a systematic review. Hum Reprod Update 27:108–29 33099635 10.1093/humupd/dmaa038

[156] Jiang X, Chen X (2023) Endometrial cell–derived exosomes facilitate the development of adenomyosis via the IL–6/JAK2/STAT3 pathway. Exp Ther Med 26:526 37869633 10.3892/etm.2023.12225 PMC10587878

[157] Matsuzaki S, Pouly JL, Canis M (2022) Persistent activation of Signal Transducer And Activator Of Transcription 3 via Interleukin-6 trans-signaling is involved in fibrosis of endometriosis. Hum Reprod 37:1489–1504 35551394 10.1093/humrep/deac098

[158] Szóstek-Mioduchowska AZ, Baclawska A, Okuda K, Skarzynski DJ (2019) Effect of proinflammatory cytokines on endometrial collagen and metallopeptidase expression during the course of equine endometrosis. Cytokine 123:154767 31265984 10.1016/j.cyto.2019.154767

[159] Lin K, Ma J, Peng Y, Sun M, Xu K, Wu R, Lin J (2019) Autocrine production of Interleukin-34 promotes the development of endometriosis through CSF1R/JAK3/STAT6 signaling. Sci Rep 9:16781 31727934 10.1038/s41598-019-52741-1 PMC6856158

[160] Ulukus M, Ulukus EC, Seval Y, Cinar O, Zheng W, Arici A (2006) Expression of Interleukin-8 receptors in patients with adenomyosis. Fertil Steril 85:714–720 16500343 10.1016/j.fertnstert.2005.08.053

[161] Ulukus EC, Ulukus M, Seval Y, Zheng W, Arici A (2005) Expression of Interleukin-8 and Monocyte Chemotactic Protein-1 in adenomyosis. Hum Reprod 20:2958–2963 15979992 10.1093/humrep/dei154

[162] Sikora J, Smycz-Kubańska M, Mielczarek-Palacz A, Kondera-Anasz Z (2017) Abnormal peritoneal regulation of chemokine activation-the role of IL-8 in pathogenesis of endometriosis. Am J Reprod Immunol. 10.1111/aji.12622 28120482 10.1111/aji.12622

[163] Borrelli GM, Abrão MS, Mechsner S (2014) Can chemokines be used as biomarkers for endometriosis? A systematic review. Hum Reprod 29:253–266 24287816 10.1093/humrep/det401

[164] Huang J, Chen X, Lv Y (2021) HMGB1 mediated inflammation and autophagy contribute to endometriosis. Front Endocrinol (Lausanne) 12:616696 33815277 10.3389/fendo.2021.616696 PMC8018282

[165] Huang J, Chen X, Liu J (2024) High mobility group box 1 promotes endometriosis under hypoxia by regulating inflammation and autophagy in vitro and in vivo. Int Immunopharmacol 127:111397 38134596 10.1016/j.intimp.2023.111397

[166] Shimizu K, Kamada Y, Sakamoto A, Matsuda M, Nakatsuka M, Hiramatsu Y (2017) High expression of high-mobility group box 1 in menstrual blood: implications for endometriosis. Reprod Sci 24:1532–1537 29017437 10.1177/1933719117692042

[167] Liu XN, Cheng ZP (2023) Expression of high-mobility group box-1 in eutopic/ectopic endometrium and correlations with inflammation-related factors in adenomyosis. Gynecol Endocrinol 39:2269265 37967572 10.1080/09513590.2023.2269265

[168] Osuga Y, Hirota Y, Yoshino O, Hirata T, Koga K, Taketani Y (2012) Proteinase-activated receptors in the endometrium and endometriosis. Front Biosci (Schol Ed) 4:1201–1212 22652866 10.2741/s326

[169] Irandoost E, Najibi S, Talebbeigi S, Nassiri S (2023) Focus on the role of NLRP3 inflammasome in the pathology of endometriosis: a review on molecular mechanisms and possible medical applications. Naunyn Schmiedebergs Arch Pharmacol 396:621–631 36542122 10.1007/s00210-022-02365-6

[170] Liu H, Zhao Y, Yang Y, Huang W, Chao L (2022) GRIM19 downregulation-induced pyroptosis of macrophages through NLRP3 pathway in adenomyosis. Reprod Biomed Online 44:211–219 34906422 10.1016/j.rbmo.2021.10.012

[171] Liu Y, Jiang Z, Zhang L, Tian W, Lin A, Li M (2024) Blockage of the NLRP3 inflammasome by MCC950 inhibits migration and invasion in adenomyosis. Reprod Biomed Online. 10.1016/j.rbmo.2024.104319 39121559 10.1016/j.rbmo.2024.104319

[172] Shi L, Xue X, Tian H, Ye H, Wang H, Wang R, Liu Y, Zhang C, Chen Q, Sun L (2021) WEE1 promotes endometriosis via the Wnt/β-catenin signaling pathway. Reprod Biol Endocrinol 19:161 34686198 10.1186/s12958-021-00844-8 PMC8532311

[173] Miyashita M, Koga K, Takeuchi A, Makabe T, Taguchi A, Urata Y, Izumi G, Takamura M, Harada M, Hirata T, Hirota Y, Wada-Hiraike O, Yoshino O, Fujii T, Osuga Y (2018) Expression of nerve injury-induced protein1 (Ninj1) in endometriosis. Reproductive Sci 26:1105–1110 10.1177/1933719118806395 30326781

[174] Carrarelli P, Yen CF, Funghi L, Arcuri F, Tosti C, Bifulco G, Luddi A, Lee CL, Petraglia F (2017) Expression of inflammatory and neurogenic mediators in adenomyosis. Reprod Sci 24:369–375 27440813 10.1177/1933719116657192

[175] Vergetaki A, Jeschke U, Vrekoussis T, Taliouri E, Sabatini L, Papakonstanti EA, Makrigiannakis A (2013) Differential expression of CRH, UCN, CRHR1 and CRHR2 in eutopic and ectopic endometrium of women with endometriosis. PLoS One 8:e62313 23638035 10.1371/journal.pone.0062313 PMC3634725

[176] Novembri R, Carrarelli P, Toti P, Rocha AL, Borges LE, Reis FM, Piomboni P, Florio P, Petraglia F (2011) Urocortin 2 and urocortin 3 in endometriosis: evidence for a possible role in inflammatory response. Mol Hum Reprod 17:587–593 21454316 10.1093/molehr/gar020

[177] Zhang H, Li C, Li W, Xin W, Qin T (2024) Research advances in adenomyosis-related signaling pathways and promising targets. Biomolecules. 10.3390/biom14111402 39595579 10.3390/biom14111402 PMC11591984

[178] González-Ramos R, Defrère S, Devoto L (2012) Nuclear factor-kappaB: a main regulator of inflammation and cell survival in endometriosis pathophysiology. Fertil Steril 98:520–528 22771029 10.1016/j.fertnstert.2012.06.021

[179] Wu M, Zhang Y (2021) MiR-182 inhibits proliferation, migration, invasion and inflammation of endometrial stromal cells through deactivation of NF-κB signaling pathway in endometriosis. Mol Cell Biochem 476:1575–1588 33400022 10.1007/s11010-020-03986-2

[180] Iba Y, Harada T, Horie S, Deura I, Iwabe T, Terakawa N (2004) Lipopolysaccharide-promoted proliferation of endometriotic stromal cells via induction of tumor necrosis factor alpha and interleukin-8 expression. Fertil Steril 82(Suppl 3):1036–1042 15474070 10.1016/j.fertnstert.2004.04.038

[181] Azuma Y, Taniguchi F, Nakamura K, Nagira K, Khine YM, Kiyama T, Uegaki T, Izawa M, Harada T (2017) Lipopolysaccharide promotes the development of murine endometriosis-like lesions via the nuclear factor-kappa B pathway. Am J Reprod Immunol. 10.1111/aji.12631 28138997 10.1111/aji.12631

[182] Ota H, Igarashi S, Hatazawa J, Tanaka T (1998) Is adenomyosis an immune disease? Hum Reprod Update 4:360–367 9825851 10.1093/humupd/4.4.360

[183] Wang F, Li H, Yang Z, Du X, Cui M, Wen Z (2009) Expression of interleukin-10 in patients with adenomyosis. Fertil Steril 91:1681–1685 18439592 10.1016/j.fertnstert.2008.02.164

[184] Feng T, Wei S, Wang Y, Fu X, Shi L, Qu L, Fan X (2017) Rhein ameliorates adenomyosis by inhibiting NF-κB and β-Catenin signaling pathway. Biomed Pharmacother 94:231–237 28763746 10.1016/j.biopha.2017.07.089

[185] Li B, Chen M, Liu X, Guo S-W (2013) Constitutive and tumor necrosis factor-α-induced activation of nuclear factor-κB in adenomyosis and its inhibition by andrographolide. Fertil Steril 100:568–577 23706331 10.1016/j.fertnstert.2013.04.028

[186] Veillat V, Lavoie CH, Metz CN, Roger T, Labelle Y, Akoum A (2009) Involvement of nuclear factor-kappaB in macrophage migration inhibitory factor gene transcription up-regulation induced by interleukin- 1 beta in ectopic endometrial cells. Fertil Steril 91:2148–2156 18710704 10.1016/j.fertnstert.2008.05.017

[187] Park H, Kim S-H, Cho YM, Ihm HJ, Oh YS, Hong SH, Chae HD, Kim C-H, Kang BM (2016) Increased expression of nuclear factor kappa-B p65 subunit in adenomyosis. Obstet Gynecol Sci 59:123–129 27004203 10.5468/ogs.2016.59.2.123 PMC4796082

[188] Lu Q, Huang Y, Wu J, Guan Y, Du M, Wang F, Liu Z, Zhu Y, Gong G, Hou H, Zhang M, Zhang JY, Ning F, Chen L, Wang L, Lash GE (2020) T-cadherin inhibits invasion and migration of endometrial stromal cells in endometriosis. Hum Reprod 35:145–156 31886853 10.1093/humrep/dez252

[189] Zhang H, Li M, Wang F, Liu S, Li J, Wen Z, Zhao X (2010) Endometriotic epithelial cells induce MMPs expression in endometrial stromal cells via an NFkappaB-dependent pathway. Gynecol Endocrinol 26:456–467 19903119 10.3109/09513590903366988

[190] Nasiri N, Babaei S, Moini A, Eftekhari-Yazdi P (2021) Controlling semi-invasive activity of human endometrial stromal cells by inhibiting NF-kB signaling pathway using Aloe-emodin and Aspirin. J Reprod Infertil 22:227–240 34987984 10.18502/jri.v22i4.7648 PMC8669405

[191] Lai TH, Wu PH, Wu WB (2016) Involvement of NADPH oxidase and NF-κB activation in CXCL1 induction by vascular endothelial growth factor in human endometrial epithelial cells of patients with adenomyosis. J Reprod Immunol 118:61–69 27665197 10.1016/j.jri.2016.08.011

[192] Wang X, Ren R, Shao M, Lan J (2020) MicroRNA–16 inhibits endometrial stromal cell migration and invasion through suppression of the inhibitor of nuclear factor–κB kinase subunit β/nuclear factor–κB pathway. Int J Mol Med 46:740–750 32626910 10.3892/ijmm.2020.4620 PMC7307865

[193] An M, Fu X, Meng X, Liu H, Ma Y, Li Y, Li Q, Chen J (2024) PI3K/AKT signaling pathway associates with pyroptosis and inflammation in patients with endometriosis. J Reprod Immunol 162:104213 38364342 10.1016/j.jri.2024.104213

[194] Zhou J, Chern BSM, Barton-Smith P, Phoon JWL, Tan TY, Viardot-Foucault V, Ku CW, Tan HH, Chan JKY, Lee YH (2020) Peritoneal fluid cytokines reveal new insights of endometriosis subphenotypes. Int J Mol Sci. 10.3390/ijms21103515 32429215 10.3390/ijms21103515 PMC7278942

[195] Jiang Y, Jiang R, Cheng X, Zhang Q, Hu Y, Zhang H, Cao Y, Zhang M, Wang J, Ding L, Diao Z, Sun H, Yan G (2016) Decreased expression of NR4A nuclear receptors in adenomyosis impairs endometrial decidualization. Mol Hum Reprod 22:655–668 27515096 10.1093/molehr/gaw042

[196] Samartzis N, Kalaitzopoulos DR, Noske A, Ihnenfeld I, Hutmacher J, Imesch P, Samartzis EP (2023) The immunohistochemical expression of GPER and classical sex hormone receptors differs in adenomyosis and eutopic endometrium. J Reprod Immunol 156:103795 36709642 10.1016/j.jri.2023.103795

[197] Kanda N, Watanabe S (2003) 17beta-estradiol inhibits oxidative stress-induced apoptosis in keratinocytes by promoting Bcl-2 expression. J Invest Dermatol 121:1500–1509 14675202 10.1111/j.1523-1747.2003.12617.x

[198] Yuan J, Liu M, Yang L, Tu G, Zhu Q, Chen M, Cheng H, Luo H, Fu W, Li Z, Yang G (2015) Acquisition of epithelial-mesenchymal transition phenotype in the Tamoxifen-resistant breast cancer cell: a new role for G protein-coupled estrogen receptor in mediating Tamoxifen resistance through cancer-associated fibroblast-derived fibronectin and β1-integrin signaling pathway in tumor cells. Breast Cancer Res 17:69 25990368 10.1186/s13058-015-0579-y PMC4453053

[199] De Francesco EM, Lappano R, Santolla MF, Marsico S, Caruso A, Maggiolini M (2013) HIF-1α/GPER signaling mediates the expression of VEGF induced by hypoxia in breast cancer associated fibroblasts (CAFs). Breast Cancer Res 15:R64 23947803 10.1186/bcr3458 PMC3978922

[200] Rigiracciolo DC, Scarpelli A, Lappano R, Pisano A, Santolla MF, De Marco P, Cirillo F, Cappello AR, Dolce V, Belfiore A, Maggiolini M, De Francesco EM (2015) Copper activates HIF-1α/GPER/VEGF signalling in cancer cells. Oncotarget 6:34158–34177 26415222 10.18632/oncotarget.5779 PMC4741443

[201] Xu XY, Zhang J, Qi YH, Kong M, Liu SA, Hu JJ (2018) Linc-ROR promotes endometrial cell proliferation by activating the PI3K-Akt pathway. Eur Rev Med Pharmacol Sci 22:2218–2225 29762822 10.26355/eurrev_201804_14807

[202] Nissinen L, Kähäri V-M (2014) Matrix metalloproteinases in inflammation. Biochim et Biophys Acta (BBA) - Gen Subj 1840:2571–2580 10.1016/j.bbagen.2014.03.007 24631662

[203] Zayratyants OV, Adamyan LV, Manukyan LM, Kalinin DV, Arslanyan KN (2018) [The expression of moesin, p21-activated kinase 4 (PAK 4), matrix metalloproteinases (MMP 2, MMP 9), and CD34 in the eutopic and ectopic endometrium in adenomyosis]. Arkh Patol 80:14–21 30585588 10.17116/patol20188006114

[204] Li T, Li YG, Pu DM (2006) Matrix metalloproteinase-2 and – 9 expression correlated with angiogenesis in human adenomyosis. Gynecol Obstet Invest 62:229–235 16837781 10.1159/000094426

[205] Spuijbroek MDEH, Dunselman GAJ, Menheere PPCA, Evers JLH (1992) Early endometriosis invades the extracellular matrix. Fertil Steril 58:929–933 1426378 10.1016/s0015-0282(16)55437-5

[206] Zhao XW, Li Y, Wang N, Zhao J, Li XL, Liu Q, Jia JH, Yang ZF, Kang S (2008) [Study on the association of SNPs of MMP-2 and TIMP-2 genes with the risk of endometriosis and adenomyosis]. Zhonghua Yi Xue Yi Chuan Xue Za Zhi 25:280–3 18543216

[207] Marbaix E, Kokorine I, Henriet P, Donnez J, Courtoy PJ, Eeckhout Y (1995) The expression of interstitial collagenase in human endometrium is controlled by progesterone and by oestradiol and is related to menstruation. Biochem J 305:1027–1030 7848264 10.1042/bj3051027 PMC1136361

[208] Yi KW, Kim SH, Ihm HJ, Oh YS, Chae HD, Kim CH, Kang BM (2015) Increased expression of p21-activated kinase 4 in adenomyosis and its regulation of matrix metalloproteinase-2 and − 9 in endometrial cells. Fertil Steril 103:1089–97.e2 25637478 10.1016/j.fertnstert.2014.12.124

[209] Yang M, Jiang C, Chen H, Nian Y, Bai Z, Ha C (2015) The involvement of osteopontin and matrix metalloproteinase- 9 in the migration of endometrial epithelial cells in patients with endometriosis. Reprod Biol Endocrinol 13:95 26289107 10.1186/s12958-015-0090-4 PMC4545920

[210] Ullah A, Wang MJ, Wang YX, Shen B (2023) CXC chemokines influence immune surveillance in immunological disorders: polycystic ovary syndrome and endometriosis. Biochim Biophys Acta Mol Basis Dis 1869:166704 37001703 10.1016/j.bbadis.2023.166704

[211] Peng Y, Ma J, Lin J (2019) Activation of the CXCL16/CXCR6 axis by TNF-α contributes to ectopic endometrial stromal cells migration and invasion. Reprod Sci 26:420–427 29779473 10.1177/1933719118776797

[212] Chand AL, Murray AS, Jones RL, Hannan NJ, Salamonsen LA, Rombauts L (2007) Laser capture microdissection and cDNA array analysis of endometrium identify CCL16 and CCL21 as epithelial-derived inflammatory mediators associated with endometriosis. Reprod Biol Endocrinol 5:18 17506907 10.1186/1477-7827-5-18 PMC1884154

[213] Aksak T, Gümürdülü D, Çetin MT, Polat S (2021) Expression of monocyte chemotactic protein 2 and tumor necrosis factor alpha in human normal endometrium and endometriotic tissues. J Gynecol Obstet Hum Reprod 50:101971 33152545 10.1016/j.jogoh.2020.101971

[214] Wang S, Duan H, Li BH, Wang YY, Huang JH, Guo ZC (2020) [Expression and significance of chemokine CXCL12 and receptor CXCR4 in adenomyosis]. Zhonghua fu chan ke za zhi 55:754–759 33228346 10.3760/cma.j.cn112141-20200226-00140

[215] Xu H, Yang Y, Zhou C, Huang X, Lin J, Zhang X (2014) Increased endometrial expression of CC-chemokine receptor-1 in women with adenomyosis. Histol Histopathol 29:1153–1160 24599574 10.14670/HH-29.1153

[216] Li J, Yin G, Chen M, Yang S, Wu A, Liang J, Yuan Z (2017) Expression of CXCL12 and its receptor CXCR4 in patients with adenomyosis. Oncol Lett 13:2731–2736 28454459 10.3892/ol.2017.5762 PMC5403167

[217] Ikebuchi A, Osaki M, Wada I, Nagata H, Nagira K, Azuma Y, Okada F, Harada T, Taniguchi F (2023) Increased chemokine ligand 26 expression and its involvement in epithelial-mesenchymal transition in the endometrium with adenomyosis. J Gynecol Obstet Hum Reprod 52:102645 37597576 10.1016/j.jogoh.2023.102645

[218] Qu H, Li L, Wang TL, Seckin T, Segars J, Shih IM (2020) Epithelial cells in endometriosis and adenomyosis upregulate STING expression. Reprod Sci 27:1276–1284 32046461 10.1007/s43032-019-00127-z PMC7539849

[219] Wang X, Wei Z, Tang Z, Xue C, Yu H, Zhang D, Li Y, Liu X, Shi Y, Zhang L, Chen G, Zhou H, Wang J, Wang X (2021) IL-37bΔ1–45 suppresses the migration and invasion of endometrial cancer cells by targeting the Rac1/NF-κB/MMP2 signal pathway. Lab Invest 101:760–74 33753880 10.1038/s41374-021-00544-2

[220] He Y, Xiong T, Guo F, Du Z, Fan Y, Sun H, Feng Z, Zhang G (2020) Interleukin-37b inhibits the growth of murine endometriosis-like lesions by regulating proliferation, invasion, angiogenesis and inflammation. Mol Hum Reprod 26:240–255 32119739 10.1093/molehr/gaaa014

[221] Jiang JF, Xiao SS, Xue M (2018) Decreased expression of interleukin-37 in the ectopic and eutopic endometria of patients with adenomyosis. Gynecol Endocrinol 34:83–86 28762845 10.1080/09513590.2017.1354367

[222] Nematian SE, Mamillapalli R, Kadakia TS, Majidi Zolbin M, Moustafa S, Taylor HS (2018) Systemic inflammation induced by microRNAs: endometriosis-derived alterations in circulating microRNA 125b-5p and Let-7b-5p regulate macrophage cytokine production. J Clin Endocrinol Metab 103:64–74 29040578 10.1210/jc.2017-01199

[223] Li L, Ye K, Wang D (2023) Upregulation of HTRA1 mediated by the lncRNA NEAT1/miR-141-3p axis contributes to endometriosis development through activating NLRP3 inflammasome-mediated pyroptotic cell death and cellular inflammation. In Vitro Cell Dev Biol Anim 59:166–178 37017808 10.1007/s11626-023-00760-8

[224] Le NXH, Loret de Mola JR, Bremer P, Groesch K, Wilson T, Diaz-Sylvester P, Braundmeier-Fleming AG (2021) Alteration of systemic and uterine endometrial immune populations in patients with endometriosis. Am J Reprod Immunol 85:e13362 33070438 10.1111/aji.13362

[225] Malvezzi H, Hernandes C, Piccinato CA, Podgaec S (2019) Interleukin in endometriosis-associated infertility-pelvic pain: systematic review and meta-analysis. Reproduction 158:1–12 30933927 10.1530/REP-18-0618

[226] Kokot I, Piwowar A, Jędryka M, Sołkiewicz K, Kratz EM (2021) Diagnostic significance of selected serum inflammatory markers in women with advanced endometriosis. Int J Mol Sci. 10.3390/ijms22052295 33669013 10.3390/ijms22052295 PMC7956504

[227] Sikora J, Mielczarek-Palacz A, Kondera-Anasz Z, Strzelczyk J (2015) Peripheral blood proinflammatory response in women during menstrual cycle and endometriosis. Cytokine 76:117–122 26315533 10.1016/j.cyto.2015.08.007

[228] Mu F, Harris HR, Rich-Edwards JW, Hankinson SE, Rimm EB, Spiegelman D, Missmer SA (2018) A prospective study of inflammatory markers and risk of endometriosis. Am J Epidemiol 187:515–522 28992341 10.1093/aje/kwx272 PMC5859972

[229] Bellelis P, Frediani Barbeiro D, Gueuvoghlanian-Silva BY, Kalil J, Abrão MS, Podgaec S (2019) Interleukin-15 and Interleukin-7 are the major cytokines to maintain endometriosis. Gynecol Obstet Invest 84:435–44 30712043 10.1159/000496607

[230] Kobayashi H (2023) Endometrial inflammation and impaired spontaneous decidualization: insights into the pathogenesis of adenomyosis. Int J Environ Res Public Health. 10.3390/ijerph20043762 36834456 10.3390/ijerph20043762 PMC9964052

[231] Zhang L, Yu Z, Qu Q, Li X, Lu X, Zhang H (2022) Exosomal lncRNA HOTAIR promotes the progression and angiogenesis of endometriosis via the miR-761/HDAC1 axis and activation of STAT3-mediated inflammation. Int J Nanomed 17:1155–1170 10.2147/IJN.S354314 PMC8935629 35321026

[232] Berbic M, Fraser IS (2011) Regulatory T cells and other leukocytes in the pathogenesis of endometriosis. J Reprod Immunol 88:149–155 21269709 10.1016/j.jri.2010.11.004

[233] Kusama K, Fukushima Y, Yoshida K, Sakakibara H, Tsubata N, Yoshie M, Kojima J, Nishi H, Tamura K (2021) Endometrial epithelial-mesenchymal transition (EMT) by menstruation-related inflammatory factors during hypoxia. Mol Hum Reprod. 10.1093/molehr/gaab036 33983443 10.1093/molehr/gaab036

[234] Björk E, Vinnars MT, Nagaev I, Nagaeva O, Lundin E, Ottander U, Mincheva-Nilsson L (2020) Enhanced local and systemic inflammatory cytokine mRNA expression in women with endometriosis evokes compensatory adaptive regulatory mRNA response that mediates immune suppression and impairs cytotoxicity. Am J Reprod Immunol 84:e13298 32623813 10.1111/aji.13298

[235] Szyllo K, Tchorzewski H, Banasik M, Glowacka E, Lewkowicz P, Kamer-Bartosinska A (2003) The involvement of T lymphocytes in the pathogenesis of endometriotic tissues overgrowth in women with endometriosis. Mediators Inflamm 12:131–138 12857596 10.1080/0962935031000134842 PMC1781609

[236] Liu Z, Sun Z, Liu H, Niu W, Wang X, Liang N, Wang X, Wang Y, Shi Y, Xu L, Shi W (2021) Single-cell transcriptomic analysis of eutopic endometrium and ectopic lesions of adenomyosis. Cell Biosci 11:51 33685511 10.1186/s13578-021-00562-z PMC7938473

[237] Greaves E, Temp J, Esnal-Zufiurre A, Mechsner S, Horne AW, Saunders PT (2015) Estradiol is a critical mediator of macrophage-nerve cross talk in peritoneal endometriosis. Am J Pathol 185:2286–2297 26073038 10.1016/j.ajpath.2015.04.012 PMC4530129

[238] Liang Y, Xie H, Wu J, Liu D, Yao S (2018) Villainous role of estrogen in macrophage-nerve interaction in endometriosis. Reprod Biol Endocrinol 16:122 30518376 10.1186/s12958-018-0441-z PMC6282253

[239] Montagna P, Capellino S, Villaggio B, Remorgida V, Ragni N, Cutolo M, Ferrero S (2008) Peritoneal fluid macrophages in endometriosis: correlation between the expression of estrogen receptors and inflammation. Fertil Steril 90:156–164 17548071 10.1016/j.fertnstert.2006.11.200

[240] Gou Y, Li X, Li P, Zhang H, Xu T, Wang H, Wang B, Ma X, Jiang X, Zhang Z (2019) Estrogen receptor β upregulates CCL2 via NF-κB signaling in endometriotic stromal cells and recruits macrophages to promote the pathogenesis of endometriosis. Hum Reprod 34:646–658 30838396 10.1093/humrep/dez019

[241] Weng L-c, Hou S-h, Lei S-t, Peng H-y, Li M-q, Zhao D (2020) Estrogen-regulated CD200 inhibits macrophage phagocytosis in endometriosis. J Reprod Immunol 138:103090 32014721 10.1016/j.jri.2020.103090

[242] McCallion A, Nasirzadeh Y, Lingegowda H, Miller JE, Khalaj K, Ahn S, Monsanto SP, Bidarimath M, Sisnett DJ, Craig AW, Young SL, Lessey BA, Koti M, Tayade C (2022) Estrogen mediates inflammatory role of mast cells in endometriosis pathophysiology. Front Immunol 13:961599 36016927 10.3389/fimmu.2022.961599 PMC9396281

[243] Li Y, Zou S, Xia X, Zhang S (2015) Human adenomyosis endometrium stromal cells secreting more nerve growth factor: impact and effect. Reprod Sci 22:1073–1082 25519715 10.1177/1933719114561559

[244] Marshall JS, Gomi K, Blennerhassett MG, Bienenstock J (1999) Nerve growth factor modifies the expression of inflammatory cytokines by mast cells via a prostanoid-dependent mechanism. J Immunol 162:4271–4276 10201958

[245] Xu X, Wang J, Guo X, Chen Y, Ding S, Zou G, Zhu L, Li T, Zhang X (2023) GPR30-mediated non-classic estrogen pathway in mast cells participates in endometriosis pain via the production of FGF2. Front Immunol 14:1106771 36845134 10.3389/fimmu.2023.1106771 PMC9945179

[246] Zhu TH, Ding SJ, Li TT, Zhu LB, Huang XF, Zhang XM (2018) Estrogen is an important mediator of mast cell activation in ovarian endometriomas. Reprod 155:73–83 10.1530/REP-17-0457 29074615

[247] Guo X, Xu X, Li T, Yu Q, Wang J, Chen Y, Ding S, Zhu L, Zou G, Zhang X (2021) NLRP3 Inflammasome Activation of Mast Cells by Estrogen via the Nuclear-Initiated Signaling Pathway Contributes to the Development of Endometriosis. Front Immunol 12:749979 34630429 10.3389/fimmu.2021.749979 PMC8494307

[248] Li YY, Lin YK, Li Y, Liu XH, Li DJ, Wang XL, Wang L, Zhu YZ, Yu M, Du MR (2022) SCM-198 alleviates endometriosis by suppressing Estrogen-ERα mediated differentiation and function of CD4(+)CD25(+) regulatory T cells. Int J Biol Sci 18:1961–1973 35342349 10.7150/ijbs.68224 PMC8935231

[249] Mei J, Li MQ, Ding D, Li DJ, Jin LP, Hu WG, Zhu XY (2013) Indoleamine 2,3-dioxygenase-1 (IDO1) enhances survival and invasiveness of endometrial stromal cells via the activation of JNK signaling pathway. Int J Clin Exp Pathol 6:431–444 23411497 PMC3563200

[250] Wei C, Mei J, Tang L, Liu Y, Li D, Li M, Zhu X (2016) 1-Methyl-tryptophan attenuates regulatory T cells differentiation due to the inhibition of estrogen-IDO1-MRC2 axis in endometriosis. Cell Death Dis 7:e2489 27906184 10.1038/cddis.2016.375 PMC5260991

[251] Yang H, Yin J, Ficarrotta K, Hsu SH, Zhang W, Cheng C (2016) Aberrant expression and hormonal regulation of Galectin-3 in endometriosis women with infertility. J Endocrinol Invest 39:785–791 26886939 10.1007/s40618-016-0435-7 PMC4906070

[252] Yamashita S, Hashimoto K, Sawada I, Ogawa M, Nakatsuka E, Kawano M, Kinose Y, Kodama M, Sawada K, Kimura T (2022) Endometrial galectin-3 causes endometriosis by supporting eutopic endometrial cell survival and engraftment in the peritoneal cavity. Am J Reprod Immunol 87:e13533 35366371 10.1111/aji.13533

[253] Hisrich BV, Young RB, Sansone AM, Bowens Z, Green LJ, Lessey BA, Blenda AV (2020) Role of human galectins in inflammation and cancers associated with endometriosis. Biomolecules. 10.3390/biom10020230 32033052 10.3390/biom10020230 PMC7072718

[254] Caserta D, Di Benedetto L, Bordi G, D’Ambrosio A, Moscarini M (2014) Levels of Galectin-3 and Stimulation Expressed Gene 2 in the peritoneal fluid of women with endometriosis: a pilot study. Gynecol Endocrinol 30:877–880 25069762 10.3109/09513590.2014.943728

[255] Kang YJ, Cho HJ, Lee Y, Park A, Kim MJ, Jeung IC, Jung YW, Jung H, Choi I, Lee HG, Yoon SR (2023) IL-17A and Th17 cells contribute to endometrial cell survival by inhibiting apoptosis and NK cell mediated cytotoxicity of endometrial cells via ERK1/2 pathway. Immune Netw 23:e14 37179747 10.4110/in.2023.23.e14 PMC10166657

[256] Ahn SH, Edwards AK, Singh SS, Young SL, Lessey BA, Tayade C (2015) IL-17A contributes to the pathogenesis of endometriosis by triggering proinflammatory cytokines and angiogenic growth factors. J Immunol 195:2591–2600 26259585 10.4049/jimmunol.1501138 PMC4561197

[257] Hirata T, Osuga Y, Hamasaki K, Yoshino O, Ito M, Hasegawa A, Takemura Y, Hirota Y, Nose E, Morimoto C, Harada M, Koga K, Tajima T, Saito S, Yano T, Taketani Y (2008) Interleukin (IL)-17A stimulates IL-8 secretion, cyclooxygensase-2 expression, and cell proliferation of endometriotic stromal cells. Endocrinology 149:1260–1267 18079209 10.1210/en.2007-0749

[258] Roberts M, Luo X, Chegini N (2005) Differential regulation of interleukins IL-13 and IL-15 by ovarian steroids, TNF-alpha and TGF-beta in human endometrial epithelial and stromal cells. Mol Hum Reprod 11:751–760 16254005 10.1093/molehr/gah233

[259] Yu JJ, Sun HT, Zhang ZF, Shi RX, Liu LB, Shang WQ, Wei CY, Chang KK, Shao J, Wang MY, Li MQ (2016) IL15 promotes growth and invasion of endometrial stromal cells and inhibits killing activity of NK cells in endometriosis. Reproduction 152:151–160 27190213 10.1530/REP-16-0089

[260] Khan KN, Kitajima M, Imamura T, Hiraki K, Fujishita A, Sekine I, Ishimaru T, Masuzaki H (2008) Toll-like receptor 4-mediated growth of endometriosis by human heat-shock protein 70. Hum Reprod 23:2210–2219 18596029 10.1093/humrep/den195

[261] Khan KN, Kitajima M, Inoue T, Tateishi S, Fujishita A, Nakashima M, Masuzaki H (2013) Additive effects of inflammation and stress reaction on Toll-like receptor 4-mediated growth of endometriotic stromal cells. Hum Reprod 28:2794–2803 23842561 10.1093/humrep/det280

[262] Yun BH, Chon SJ, Choi YS, Cho S, Lee BS, Seo SK (2016) Pathophysiology of endometriosis: Role of High Mobility Group Box-1 and Toll-Like Receptor 4 developing inflammation in endometrium. PLoS One 11:e0148165 26872033 10.1371/journal.pone.0148165 PMC4752230

[263] Khan KN, Kitajima M, Inoue T, Fujishita A, Nakashima M, Masuzaki H (2015) 17β-estradiol and lipopolysaccharide additively promote pelvic inflammation and growth of endometriosis. Reprod Sci 22:585–594 25355803 10.1177/1933719114556487 PMC4519769

[264] Dantzler MD, Miller TA, Dougherty MW, Quevedo A (2025) The microbiome landscape of adenomyosis: a systematic review. Reprod Sci 32:251–260 39707139 10.1007/s43032-024-01766-7

[265] Ferrero S, Remorgida V, Maganza C, Venturini PL, Salvatore S, Papaleo E, Candiani M, Leone Roberti Maggiore U (2014) Aromatase and endometriosis: estrogens play a role. Ann N Y Acad Sci 1317:17–23 24738993 10.1111/nyas.12411

[266] Bulun SE, Gurates B, Fang Z, Tamura M, Sebastian S, Zhou J, Amin S, Yang S (2002) Mechanisms of excessive estrogen formation in endometriosis. J Reprod Immunol 55:21–33 12062819 10.1016/s0165-0378(01)00132-2

[267] Maia H Jr., Casoy J, Valente Filho J (2009) Is aromatase expression in the endometrium the cause of endometriosis and related infertility? Gynecol Endocrinol 25:253–7 19340622 10.1080/09513590802627647

[268] Tsai SJ, Wu MH, Lin CC, Sun HS, Chen HM (2001) Regulation of steroidogenic acute regulatory protein expression and progesterone production in endometriotic stromal cells. J Clin Endocrinol Metab 86:5765–5773 11739437 10.1210/jcem.86.12.8082

[269] Sacco K, Portelli M, Pollacco J, Schembri-Wismayer P, Calleja-Agius J (2012) The role of prostaglandin E2 in endometriosis. Gynecol Endocrinol 28:134–8 22003899 10.3109/09513590.2011.588753

[270] De Leon FD, Vijayakumar R, Brown M, Rao CV, Yussman MA, Schultz G (1986) Peritoneal fluid volume, estrogen, progesterone, prostaglandin, and epidermal growth factor concentrations in patients with and without endometriosis. Obstet Gynecol 68:189–194 3488529

[271] Urata Y, Osuga Y, Akiyama I, Nagai M, Izumi G, Takamura M, Hasegawa A, Harada M, Hirata T, Hirota Y, Yoshino O, Koga K, Kozuma S (2013) Interleukin-4 and prostaglandin E2 synergistically up-regulate 3β-hydroxysteroid dehydrogenase type 2 in endometrioma stromal cells. J Clin Endocrinol Metab 98:1583–1590 23450050 10.1210/jc.2012-3475

[272] Zhang H, Zhao X, Liu S, Li J, Wen Z, Li M (2010) 17betaE2 promotes cell proliferation in endometriosis by decreasing PTEN via NFkappaB-dependent pathway. Mol Cell Endocrinol 317:31–43 19932734 10.1016/j.mce.2009.11.009

[273] Reis FM, Petraglia F, Taylor RN (2013) Endometriosis: hormone regulation and clinical consequences of chemotaxis and apoptosis. Hum Reprod Update 19:406–418 23539633 10.1093/humupd/dmt010 PMC3682670

[274] Qi Q, Liu X, Zhang Q, Guo SW (2020) Platelets induce increased estrogen production through NF-κB and TGF-β1 signaling pathways in endometriotic stromal cells. Sci Rep 10:1281 31992765 10.1038/s41598-020-57997-6 PMC6987096

[275] Chen P, Wang DB, Liang YM (2016) Evaluation of estrogen in endometriosis patients: regulation of GATA-3 in endometrial cells and effects on Th2 cytokines. J Obstet Gynaecol Res 42:669–677 26890586 10.1111/jog.12957

[276] Sofic A, Husic-Selimovic A, Carovac A, Jahic E, Smailbegovic V, Kupusovic J (2016) The significance of MRI evaluation of the uterine junctional zone in the early diagnosis of adenomyosis. Acta Inf Med 24:103–106 10.5455/aim.2016.24.103-106 PMC4851503 27147800

[277] Della Corte L, Barra F, Mercorio A, Evangelisti G, Rapisarda AMC, Ferrero S, Bifulco G, Giampaolino P (2020) Tolerability considerations for gonadotropin-releasing hormone analogues for endometriosis. Expert Opin Drug Metab Toxicol 16:759–68 32597340 10.1080/17425255.2020.1789591

[278] Franke HR, van de Weijer PH, Pennings TM, van der Mooren MJ (2000) Gonadotropin-releasing hormone agonist plus “add-back” hormone replacement therapy for treatment of endometriosis: a prospective, randomized, placebo-controlled, double-blind trial. Fertil Steril 74:534–9 10973651 10.1016/s0015-0282(00)00690-7

[279] Steingold KA, Cedars M, Lu JK, Randle D, Judd HL, Meldrum DR (1987) Treatment of endometriosis with a long-acting gonadotropin-releasing hormone agonist. Obstet Gynecol 69:403–411 2950349

[280] Khan KN, Kitajima M, Hiraki K, Fujishita A, Sekine I, Ishimaru T, Masuzaki H (2010) Changes in tissue inflammation, angiogenesis and apoptosis in endometriosis, adenomyosis and uterine myoma after GnRH agonist therapy. Hum Reprod 25:642–653 20008888 10.1093/humrep/dep437

[281] Taylor HS, Giudice LC, Lessey BA, Abrao MS, Kotarski J, Archer DF, Diamond MP, Surrey E, Johnson NP, Watts NB, Gallagher JC, Simon JA, Carr BR, Dmowski WP, Leyland N, Rowan JP, Duan WR, Ng J, Schwefel B, Thomas JW, Jain RI, Chwalisz K (2017) Treatment of endometriosis-associated pain with elagolix, an oral GnRH antagonist. N Engl J Med 377:28–40 28525302 10.1056/NEJMoa1700089

[282] Muneyyirci-Delale O, Archer DF, Owens CD, Barnhart KT, Bradley LD, Feinberg E, Gillispie V, Hurtado S, Kim JH, Wang A, Wang H, Stewart EA (2021) Efficacy and safety of elagolix with add-back therapy in women with uterine fibroids and coexisting adenomyosis. F S Rep 2:338–46 34553161 10.1016/j.xfre.2021.05.004 PMC8441572

[283] Dababou S, Garzon S, Laganà AS, Ferrero S, Evangelisti G, Noventa M, D’Alterio MN, Palomba S, Uccella S, Franchi M, Barra F (2021) Linzagolix: a new GnRH-antagonist under investigation for the treatment of endometriosis and uterine myomas. Expert Opin Investig Drugs 30:903–911 34278887 10.1080/13543784.2021.1957830

[284] Donnez J, Donnez O, Dolmans MM (2023) Evolution of uterine adenomyosis volume during and after GnRH antagonist (linzagolix) treatment: lessons for further clinical trials. Fertil Steril 120:1071–1073 37495010 10.1016/j.fertnstert.2023.07.012

[285] Donnez J, Donnez O, Brethous M, Bestel E, Garner E, Charpentier S, Humberstone A, Loumaye E (2022) Treatment of symptomatic uterine adenomyosis with linzagolix, an oral gonadotrophin-releasing hormone antagonist: a pilot study. Reprod Biomed Online 44:200–203 34799277 10.1016/j.rbmo.2021.09.019

[286] Badawy AM, Elnashar AM, Mosbah AA (2012) Aromatase inhibitors or gonadotropin-releasing hormone agonists for the management of uterine adenomyosis: a randomized controlled trial. Acta Obstet Gynecol Scand 91:489–495 22229256 10.1111/j.1600-0412.2012.01350.x

[287] Kimura F, Takahashi K, Takebayashi K, Fujiwara M, Kita N, Noda Y, Harada N (2007) Concomitant treatment of severe uterine adenomyosis in a premenopausal woman with an aromatase inhibitor and a gonadotropin-releasing hormone agonist. Fertil Steril 87:1468e.e9–12 10.1016/j.fertnstert.2006.09.010 17222833

[288] Ferrero S, Venturini PL, Gillott DJ, Remorgida V (2011) Letrozole and norethisterone acetate versus letrozole and triptorelin in the treatment of endometriosis related pain symptoms: a randomized controlled trial. Reprod Biol Endocrinol 9:88 21693037 10.1186/1477-7827-9-88 PMC3141645

[289] Acién P, Velasco I, Acién M (2021) Anastrozole and levonorgrestrel-releasing intrauterine device in the treatment of endometriosis: a randomized clinical trial. BMC Womens Health 21:211 34016111 10.1186/s12905-021-01347-9 PMC8138989

[290] Machado DE, Alessandra-Perini J, Menezes de Mendonça E, Claudino MC, Nasciutti LE, Sola-Penna M, Zancan P, Alessandra Perini J (2020) Clotrimazole reduces endometriosis and the estrogen concentration by downregulating aromatase. Reproduction 159:779–86 32240980 10.1530/REP-19-0502

[291] Stratopoulou CA, Donnez J, Dolmans MM (2021) Conservative management of uterine adenomyosis: medical vs. surgical approach. J Clin Med. 10.3390/jcm10214878 34768397 10.3390/jcm10214878 PMC8584979

[292] Hirata T, Izumi G, Takamura M, Saito A, Nakazawa A, Harada M, Hirota Y, Koga K, Fujii T, Osuga Y (2014) Efficacy of dienogest in the treatment of symptomatic adenomyosis: a pilot study. Gynecol Endocrinol 30:726–729 24905725 10.3109/09513590.2014.926882

[293] Osuga Y, Watanabe M, Hagino A (2017) Long-term use of dienogest in the treatment of painful symptoms in adenomyosis. J Obstet Gynaecol Res 43:1441–1448 28737239 10.1111/jog.13406

[294] Osuga Y, Hayashi K, Kanda S (2020) Long-term use of dienogest for the treatment of primary and secondary dysmenorrhea. J Obstet Gynaecol Res 46:606–617 32050307 10.1111/jog.14209

[295] Stratton P, Hartog B, Hajizadeh N, Piquion J, Sutherland D, Merino M, Lee YJ, Nieman LK (2000) A single mid-follicular dose of CDB-2914, a new antiprogestin, inhibits folliculogenesis and endometrial differentiation in normally cycling women. Hum Reprod 15:1092–1099 10783359 10.1093/humrep/15.5.1092

[296] Gracia M, Alcalà M, Ferreri J, Rius M, Ros C, Saco MA, Martínez-Zamora M, Carmona F (2018) Ulipristal Acetate improves clinical symptoms in women with adenomyosis and uterine myomas. J Minim Invasive Gynecol 25:1274–1280 29626678 10.1016/j.jmig.2018.04.002

[297] Capmas P, Brun JL, Legendre G, Koskas M, Merviel P, Fernandez H (2021) Ulipristal acetate use in adenomyosis: a randomized controlled trial. J Gynecol Obstet Hum Reprod 50:101978 33186772 10.1016/j.jogoh.2020.101978

[298] Iacovides S, Avidon I, Baker FC (2015) What we know about primary dysmenorrhea today: a critical review. Hum Reprod Update 21:762–778 26346058 10.1093/humupd/dmv039

[299] Spitz IM, Grunberg SM, Chabbert-Buffet N, Lindenberg T, Gelber H, Sitruk-Ware R (2005) Management of patients receiving long-term treatment with mifepristone. Fertil Steril 84:1719–1726 16359971 10.1016/j.fertnstert.2005.05.056

[300] Li A, Felix JC, Yang W, Xiong DW, Minoo P, Jain JK (2007) Effect of mifepristone on endometrial matrix metalloproteinase expression and leukocyte abundance in new medroxyprogesterone acetate users. Contraception 76:57–6517586139 10.1016/j.contraception.2007.03.005

[301] Che X, Wang J, Sun W, He J, Wang Q, Zhu D, Zhu W, Zhang J, Dong J, Xu J, Zheng F, Zhou J, Zhao W, Lin Q, Ye L, Zhao X, Xu Z, Chen Y, Wang J, Wu W, Zhai L, Zhou Y, Zheng J, Zhang X (2023) Effect of Mifepristone vs placebo for treatment of adenomyosis with pain symptoms: a randomized clinical trial. JAMA Netw Open 6:e2317860 37307001 10.1001/jamanetworkopen.2023.17860 PMC10261993

[302] Wang Y, Jiang X, Wang S (2014) The influence of mifepristone to caspase 3 expression in adenomyosis. Clin Exp Obstet Gynecol 41:154–157 24779241

[303] Rivera R, Yacobson I, Grimes D (1999) The mechanism of action of hormonal contraceptives and intrauterine contraceptive devices. Am J Obstet Gynecol 181:1263–1269 10561657 10.1016/s0002-9378(99)70120-1

[304] Maia H Jr., Haddad C, Pinheiro N, Casoy J (2013) The effect of oral contraceptives on aromatase and Cox-2 expression in the endometrium of patients with idiopathic menorrhagia or adenomyosis. Int J Womens Health 5:293–9 23788841 10.2147/IJWH.S45093 PMC3684227

[305] Shaaban OM, Ali MK, Sabra AM, Abd El Aal DE (2015) Levonorgestrel-releasing intrauterine system versus a low-dose combined oral contraceptive for treatment of adenomyotic uteri: a randomized clinical trial. Contraception 92:301–307 26071673 10.1016/j.contraception.2015.05.015

[306] Hassanin AI, Youssef AA, Yousef AM, Ali MK (2021) Comparison of dienogest versus combined oral contraceptive pills in the treatment of women with adenomyosis: a randomized clinical trial. Int J Gynaecol Obstet 154:263–269 33454995 10.1002/ijgo.13600

[307] Du L, Du DH, Chen B, Ding Y, Zhang T, Xiao W (2020) Anti-inflammatory activity of Sanjie Zhentong Capsule assessed by network pharmacology analysis of adenomyosis treatment. Drug Des Devel Ther 14:697–713 32109994 10.2147/DDDT.S228721 PMC7039068

[308] Kim SY, Koo BN, Shin CS, Ban M, Han K, Kim MD (2016) The effects of single-dose dexamethasone on inflammatory response and pain after uterine artery embolisation for symptomatic fibroids or adenomyosis: a randomised controlled study. BJOG 123:580–587 26667403 10.1111/1471-0528.13785

[309] Zhu B, Chen Y, Guo M, Zhang C, Huang L, Pan Q, Lin T, Lu Y, Shen X, Zhang H (2022) Berberine attenuates hyperalgesia in mice with adenomyosis. Arch Gynecol Obstet 306:115–125 35230500 10.1007/s00404-022-06438-y

[310] Cirillo M, Argento FR, Becatti M, Fiorillo C, Coccia ME, Fatini C (2023) Mediterranean diet and oxidative stress: a relationship with pain perception in endometriosis. Int J Mol Sci. 10.3390/ijms241914601 37834048 10.3390/ijms241914601 PMC10572576

[311] Hough B, Drever N, Manger S (2026) What is the Evidence on Lifestyle Interventions for the Symptom Management of Pelvic Pain in Women With Endometriosis or Adenomyosis? A Scoping Review. Am J Lifestyle Med : 15598276261419770 10.1177/15598276261419770 PMC12935590 41767335

